# Investigation of the functional impact of CHED- and FECD4-associated *SLC4A11* mutations in human corneal endothelial cells

**DOI:** 10.1371/journal.pone.0296928

**Published:** 2024-01-22

**Authors:** Doug D. Chung, Angela C. Chen, Charlene H. Choo, Wenlin Zhang, Dominic Williams, Christopher G. Griffis, Paul Bonezzi, Kavya Jatavallabhula, Alapakkam P. Sampath, Anthony J. Aldave

**Affiliations:** Department of Ophthalmology, Stein Eye Institute at UCLA, Los Angeles, California, United States of America; National Eye Institute, UNITED STATES

## Abstract

Mutations in the solute linked carrier family 4 member 11 (*SLC4A11)* gene are associated with congenital hereditary endothelial dystrophy (CHED) and Fuchs corneal endothelial dystrophy type 4 (FECD4), both characterized by corneal endothelial cell (CEnC) dysfunction and/or cell loss leading to corneal edema and visual impairment. In this study, we characterize the impact of CHED-/FECD4-associated *SLC4A11* mutations on CEnC function and SLC4A11 protein localization by generating and comparing human CEnC (hCEnC) lines expressing wild type SLC4A11 (SLC4A11^WT^) or mutant *SLC4A11* harboring CHED-/FECD4-associated SLC4A11 mutations (SLC4A11^MU^). SLC4A11^WT^ and SLC4A11^MU^ hCEnC lines were generated to express either SLC4A11 variant 2 (V2^WT^ and V2^MU^) or variant 3 (V3^WT^ and V3^MU^), the two major variants expressed in ex vivo hCEnC. Functional assays were performed to assess cell barrier, proliferation, viability, migration, and NH_3_-induced membrane conductance. We demonstrate SLC4A11^-/-^ and SLC4A11^MU^ hCEnC lines exhibited increased migration rates, altered proliferation and decreased cell viability compared to *SLC4A11*^WT^ hCEnC. Additionally, SLC4A11^-/-^ hCEnC demonstrated decreased cell-substrate adhesion and membrane capacitances compared to SLC4A11^WT^ hCEnC. Induction with 10mM NH_4_Cl led SLC4A11^WT^ hCEnC to depolarize; conversely, SLC4A11^-/-^ hCEnC hyperpolarized and the majority of SLC4A11^MU^ hCEnC either hyperpolarized or had minimal membrane potential changes following NH_4_Cl induction. Immunostaining of primary hCEnC and SLC4A11^WT^ hCEnC lines for SLC4A11 demonstrated predominately plasma membrane staining with poor or partial colocalization with mitochondrial marker COX4 within a subset of punctate subcellular structures. Overall, our findings suggest CHED-associated *SLC4A11* mutations likely lead to hCEnC dysfunction, and ultimately CHED, by interfering with cell migration, proliferation, viability, membrane conductance, barrier function, and/or cell surface localization of the SLC4A11 protein in hCEnC. Additionally, based on their similar subcellular localization and exhibiting similar cell functional profiles, protein isoforms encoded by *SLC4A11* variant 2 and variant 3 likely have highly overlapping functional roles in hCEnC.

## Introduction

Congenital hereditary endothelial dystrophy (CHED) (Online Mendelian Inheritance in Man [OMIM] #217700) and Fuchs corneal endothelial dystrophy (FECD, OMIM #613267) belong to a group of inherited disorders that are characterized by bilateral corneal endothelial cell (CEnC) dysfunction and/or cell loss leading to corneal edema and loss of corneal clarity [1]. With an onset at birth or in early childhood, CHED has an autosomal recessive inheritance pattern and is associated with over 90 distinct mutations in the solute linked carrier family 4 member 11 (*SLC4A11)* gene, in which approximately 80 percent of affected individuals harbor homozygous or compound heterozygous mutations [2–20]. FECD is a complex disease that is associated with both environmental and genetic factors, with a typical onset in the fifth decade of life, though cases of early onset FECD are also observed [21, 22]. Demonstrating locus heterogeneity, FECD has been associated with mutations in *COL8A2* (associated with early onset FECD), *TCF4*, *ZEB1*, *AGBL1* and *SLC4A11*. Over 30 presumed pathogenic heterozygous *SLC4A11* mutations have been associated with FECD type 4 (FECD4) (OMIM #613268) [19, 23–28]. While the association of *SLC4A11* mutations with CHED and FECD4 is well-documented, the pathomechanisms by which *SLC4A11* mutations cause CHED and FECD4 are not well understood. Additionally, it is unclear how CHED- and FECD4- associated *SLC4A11* mutations lead to two distinct corneal dystrophies, and it is unknown whether the mutations have diverging impacts on SLC4A11 function or whether the zygosity of *SLC4A11* mutations is primarily what determines the type of dystrophy, CHED or FECD4.

*SLC4A11* is highly expressed in the corneal endothelium and encodes a cell-surface membrane protein with 14 transmembrane domains, extracellular loop glycosylation sites, and hydrophilic N-terminal and C-terminal cytoplasmic domains [29, 30]. Characterized as an electrogenic membrane transporter that functions as either an ammonia (NH_3_)-sensitive H^+^ transporter or NH_3_:H^+^ co-transporter, SLC4A11 exhibits enhanced inward H^+^ flux when perfused with NH_4_Cl [31, 32]. While still currently debated, SLC4A11 is hypothesized to have multiple roles in CEnC [33], which include: playing a role in transmembrane water flux; [34] facilitating glutamine catabolism to reduce ammonia-related oxidative stress in CEnC; [35–37] functioning as an ammonium-activated mitochondrial uncoupler to prevent mitochondrial membrane potential hyperpolarization and reduce superoxide production; [38] promoting endothelial pump activity by facilitating lactate:H+ flux across the corneal endothelium to maintain corneal clarity; [39, 40] aiding in cell adhesion to the Descemet membrane via its extracellular loops; [41] and functioning as an NRF2-activated oxidative stress response protein via NRF2-binding sites within the *SLC4A11* promoter [42–44].

Three *SLC4A11* transcript variants (variants 1–3) have been identified and are predicted to encode distinct SLC4A11 protein isoforms. Divergent findings regarding the relative levels of expression for the three *SLC4A11* transcript variants in human corneal endothelium have been reported, with Kao and colleagues demonstrating by qPCR analysis that variant 3 is the major *SLC4A11* transcript variant expressed, followed by variant 1, and Malhotra and colleagues demonstrating by qPCR and immunoblotting that variant 2 is the most abundantly expressed variant, followed by variant 3 [33, 45]. Differing in sequences at the N-terminal only, the SLC4A11 protein isoforms that are encoded by variant 2 and variant 3 are 891 and 875 amino acids in length, respectively, with the sequence of the last 861 amino acids of each isoform being identical. When the H^+^ transport and water flux activity in HEK293 cells that were transfected with variant 2 and variant 3 were compared, no differences in transport function were observed between the variants [33, 45]. However, the cellular functions facilitated by variant 2 and variant 3 in CEnC have yet to be characterized and compared. Furthermore, while the SLC4A11 protein isoform encoded by variant 2 has been characterized to primarily localize to the cell surface, more recent findings have indicated that a subset of expressed SLC4A11 protein in CEnC also resides within mitochondrial membranes, which raises the question of whether the N-terminal sequence differences between variant 2 and variant 3 could dictate the specific targeting of SLC4A11 to the plasma membrane and/or the membranes of intracellular organelles [38, 45].

To investigate the functional roles of *SLC4A11* variant 2 and 3 in CEnC, we generated stable human CEnC (hCEnC) lines expressing either wild type *SLC4A11* variant 2 or variant 3 and compared them to *SLC4A11*^-/-^ empty hCEnC that were generated by CRISPR-Cas9-mediated gene editing. Furthermore, to study the impact of *SLC4A11* mutations on hCEnC function and SLC4A11 subcellular localization, we generated stable hCEnC lines expressing CHED- and FECD4-associated *SLC4A11* mutations, thereby creating cell-based models of CHED and FECD4. Then, we performed various cell-based functional assays and localization studies to determine the impact of each *SLC4A11* mutation on hCEnC proliferation, migration, viability, barrier function, electrophysiological membrane response, and SLC4A11 protein localization in hCEnC.

## Material and methods

### Statement on the use of tissue from human subjects

Informed written consent was obtained from all human subjects according to the tenets of the Declaration of Helsinki. Cadaveric corneal tissue for research purposes was obtained from eye banks in the United States, and donor tissue procurement and processing adhered to the guidelines set by the Eye Bank Association of America.

### Primary and immortalized corneal endothelial cell cultures

Primary hCEnC isolation and cell culture were performed using the methods previously described [46]. In brief, the Descemet membrane was stripped from the stroma of cadaveric corneas, and then treated with collagenase A to dissociate the endothelial cells from the Descemet membrane. Then, the dissociated endothelial cells were seeded onto collagen IV coated culture plastic in Endothelial SFM (M5) medium (supplemented with fetal bovine serum, penicillin, streptomycin and amphotericin-B), followed by culturing in a 1:1 mixture of F12-Ham’s and M199 (M4) medium (supplemented with fetal bovine serum, L-ascorbic acid-2-phosphate, penicillin, streptomycin, insulin-transferrin-selenium, amphotericin-B and recombinant human bFGF). When the endothelial cells reached confluence, the medium was reverted back to M5 medium for a minimum of 10 days to restore and maintain the corneal endothelial cell state. Cell passaging was performed with TrypLE Select (Thermo Fisher Scientific).

An immortalized human corneal endothelial cell line, HCEnC-21T cells, was used to generate the *SLC4A11* wild type and mutant CEnC lines in this study, which were maintained and grown in a 1:1 mixture of F12-Ham’s medium and M199 medium, supplemented with 5% fetal bovine serum (Corning), 20 μg/mL human recombinant insulin (Thermo Fisher Scientific), 20 μg/mL ascorbic acid (Sigma-Aldrich), 10 ng/mL recombinant human fibroblast growth factor (basic), 100 μg/mL penicillin (Thermo Fisher Scientific), and 100 μg/mL streptomycin (Thermo Fisher Scientific). HEK293T cells (Thermo Fisher Scientific) were used to generate *SLC4A11* wild type and mutant lentiviruses, and were cultured in DMEM supplemented with 10% fetal bovine serum (Corning), 100 μg/mL penicillin (Thermo Fisher Scientific), and 100 μg/mL streptomycin (Thermo Fisher Scientific). Primary hCEnC and hCEnC lines were maintained in a humidified incubator containing 5% CO_2_ at 37°C.

### Generation of *SLC4A11*^-/-^ human corneal endothelial cell line using CRISPR-Cas9 mediated gene editing

While HCEnC-21T cells express only minimal levels of *SLC4A11*, in order to fully silence *SLC4A11* expression, CRISPR-Cas9 mediated gene editing of HCEnC-21T cells was employed to generate a stable *SLC4A11*^-/-^ hCEnC line. Guide RNAs (gRNA) were designed to target the Cas9 nuclease to exon 1 of *SLC4A11* transcript variant 2 (NM_032034.3), which is the first exon shared between all three major *SLC4A11* transcript variants (variants 1–3), thereby ensuring each of the major isoforms of the encoded SLC4A11 protein would be disrupted. The design tool available at crispr.MIT.edu was used to identify optimal target sequences for the gRNA with minimal potential for off-target effects. Based on the quality scores given by crispr.MIT.edu, the sequence 5’-GTCGCAGAATGGATACTTCG-3’ was selected to create the *SLC4A11* knockout gRNA (Sigma-Aldrich), which was hybridized to a complementary strand and was cloned into the pSpCas9(BB)-2A-Puro construct (Addgene plasmid #62988) containing a puromycin-resistance expression cassette [47]. The generated CRISPR-Cas9 gRNA constructs were transfected into HCEnC-21T cells using Lipofectamine LTX and Plus reagent (Thermo Fisher Scientific), and selection for successfully transfected cells was performed by dosing the media with puromycin (Thermo Fisher Scientific) at 10 μg/mL. Limiting dilutions at 0.2 cells/well in 96-well plates were performed to isolate transfected single cells that proliferated into colonies of subclones, which were identified by microscopy, then transferred to 6-well plates and grown to confluence. Genomic DNA and protein lysates were isolated from each subclone. Sanger sequencing (Laragen, Inc.) was performed on each subclone and sequences were analyzed by CRISP-ID to identify subclones with disruption of both *SLC4A11* alleles by the introduction of homozygous or compound heterozygous indels leading to premature stop codons, thereby generating *SLC4A11*^-/-^ hCEnC lines [48]. Clones that exhibited compound heterozygous indels at the targeted exon underwent allele-specific cloning to provide an unambiguous method for identifying the specific indel at each allele. Guide RNA, sequencing, and cloning primers are provided in S1 Table. To confirm the absence of SLC4A11 protein expression in the generated *SLC4A11*^-/-^ hCEnC lines, immunoblotting was performed to measure SLC4A11 protein levels using an affinity purified anti-SLC4A11 custom polyclonal rabbit antibody (provided by the Ira Kurtz Lab) (1:500 dilution) and previously characterized to recognize the human SLC4A11 carboxy terminus common to each of the three major SLC4A11 isoforms (S1 Fig) [33, 49, 50]. GAPDH was used as a loading control and measured by a mouse anti-GAPDH antibody (EMD Millipore; AB_2107445) (1:50,000 dilution).

### Generation of stable human corneal endothelial cell lines expressing wild type *SLC4A11* variant 2 or variant 3

Commercially available lentiviral transfer plasmids (pEZ-Lv201) containing full-length cDNA from *SLC4A11* variant 2 (NM_032034) (GeneCopoeia; EX-E0098-Lv201), *SLC4A11* variant 3 (NM_001174089) (GeneCopoeia; EX-E3062-Lv201), or an empty vector (GeneCopoeia; pEZ-Lv201) were used to produce *SLC4A11* V2 ^WT^, *SLC4A11* V3 ^WT^, and empty lentivirus, respectively. Each lentiviral transfer plasmid, also containing GFP and puromycin-resistance gene cassettes, was transfected into HEK293T cells with a third-generation packaging system using Lipofectamine LTX transfection with Plus reagent (Thermo Fisher Scientific) in antibiotic-free medium. Two days post transfection, viral supernatants were collected and then cleared of large particulates by centrifugation at 3000 RPM in a swinging bucket rotor (Beckman Coulter) followed by filtration through a 0.45 μm syringe filter (Thermo Fisher Scientific). To concentrate the viral particles, the viral particles in the filtered supernatant were pelleted by ultra-centrifugation at 25,000 RPM for 90 minutes at 4°C using a SW28 rotor (Beckman Coulter), and then resuspended in 25μL– 50μL DPBS for every 10mL of viral supernatant. Viral particle concentrations (VP/mL) were measured by p24 ELISAs (UCLA Integrated Molecular Technologies Core), and transduction units (TU) per mL were assessed by determining the proportion of GFP-expressing HCEnC-21T cells transduced with serial dilutions of each viral batch.

*SLC4A11*^-/-^ hCEnC generated by CRISPR-Cas9 mediated gene editing (described above) were transduced in a 12 well culture-grade plate with *SLC4A11* V2 ^WT^, *SLC4A11* V3 ^WT^, or empty lentivirus at a multiplicity of infection (MOI) value of 10 with the addition of 8μg/mL hexadimethrine bromide (Sigma-Aldrich) to facilitate infection. Three days after transduction, cells were transferred to a T-25 flask and expanded. After three weeks of puromycin drug pressure (10 μg/mL in media), limiting dilutions at 0.2 cells/well were performed in 96 well plates to isolate stably transduced subclones originating from a single cell, which were identified by detecting GFP expression using fluorescence microscopy. GFP-expressing transgenic hCEnC subclones were transferred to 6 well plates and grown to confluence. Genomic DNA and protein lysates were isolated from each candidate subclone and Sanger sequencing (Laragen, Inc.) was performed to confirm the stable integration of *SLC4A11* V2, *SLC4A11* V3, or empty transfer plasmid. Capillary-based immunoblotting was performed to validate the expression or non-expression of SLC4A11 in each candidate subclone. Once validated by Sanger sequencing and immunoblotting, three biological cell line replicates of *SLC4A11*^-/-^ hCEnC transduced with *SLC4A11* V2^WT^ or *SLC4A11* V3^WT^ cDNA (SLC4A11 V2^WT^ hCEnC and SLC4A11 V3^WT^ hCEnC, respectively), and four biological cell line replicates of *SLC4A11*^-/-^ hCEnC transduced with an empty vector (SLC4A11^-/-^ empty hCEnC) were chosen for this study.

### Generation of stable human corneal endothelial cell lines expressing mutant *SLC4A11* variant 2 and variant 3

Site-directed mutagenesis was performed on a commercially-obtained lentiviral transfer plasmid (pEZ-Lv201) containing full-length cDNA from *SLC4A11* variant 2 (NM_032034) (GeneCopoeia; EX-E0098-Lv201) using the QuickChange Lightning Site-Directed Mutagenesis Kit (Agilent Technologies; Cat # 210518–5) to introduce three CHED-associated (c.374G>A (p.Arg125His), c.1813C>T (p.Arg605X), c.2263C>T (p.Arg755Trp)) and one FECD4-associated (c.2224G>A (p.Gly742Arg)) *SLC4A11* mutations into the *SLC4A11* transcript variant 2 cDNA. Site-directed mutagenesis was also performed on commercially-purchased *SLC4A11* variant 3 lentiviral transfer plasmid (NM_001174089) (GeneCopoeia; EX-E3062-Lv201) to introduce CHED-associated (c.326G>A (p.Arg109His), c.1765C>T (p.Arg589X), c.2215C>T (p.Arg739Trp)) and FECD4-associated (c.2176G>A (p.Gly726Arg)) mutations into *SLC4A11* transcript variant 3 cDNA, which are the equivalent variant-specific mutations to the four *SLC4A11* mutations introduced in variant 2. The FECD4-associated *SLC4A11* mutation, c.2224G>A (along with its variant 3 equivalent), was chosen for this study as it demonstrated segregation in a three-generational family affected with FECD4 in an autosomal dominant pattern and as it was never previously reported with CHED to our knowledge [24]. The successful introduction of each *SLC4A11* mutation was validated by Sanger sequencing. Site-directed mutagenesis primers and validation primers are provided in S1 Table.

Stable SLC4A11 V2^MU^ and V3^MU^ hCEnC lines were created by transducing CRISPR-Cas9 engineered *SLC4A11*^-/-^ hCEnC with validated mutant *SLC4A11* variant 2 and variant 3 lentiviral transfer plasmids and selecting for stably-integrated SLC4A11 mutant hCEnC subclones using the same methods described above to generate the stable SLC4A11 V2^WT^ and V3^WT^ hCEnC lines.

### Cell proliferation and viability assays

To measure cell proliferation, batches of multiple 96 well microplates were seeded with 2,000 cells per well with 100μL of F99 media, and then incubated in a CO_2_ incubator at 37°C. Each microplate contained identical seeding conditions and replicates from each cell group. Over the next four days, at each 24-hour interval, a subset of microplates from each batch were incubated with 0.5mg/mL MTT [3-(4,5-dimethylthiazol-2-yl)-2,5-diphenyltetrazolium bromide] in 50μL of serum-free, phenol-red free DMEM/F12 (1:1) media in each well for 4 hours at 37°C. MTT formazan crystals produced by the viable cells in each well were dissolved by adding 100μL of DMSO, followed by orbital shaking for 15 minutes and gentle pipetting. Absorbances of each well were measured at 590-595nM on a microplate reader (FilterMax F5, Molecular Devices). Background absorbances were determined by measuring the absorbances of wells with MTT-supplemented media and DMSO, but absent of cells.

To determine cell viability, 20,000 cells from each group/condition were seeded in replicates into wells of 96 well microplates with 100μL of F99 media and allowed to settle for 2 hours at 37°C. Afterwards, the cells were incubated with various concentrations of tert-butyl hydroperoxide (tBH) in F99 media for 2.5 hours, followed by a PBS wash to remove the tBH, and then incubated in 50μL of serum-free, phenol-red free DMEM/F12 (1:1) media supplemented with 0.5mg/mL MTT for 4 hours. The levels of MTT formazan crystals produced by the proportion of viable cells in each well were assessed by dissolving the crystals with 100μL of DMSO and measuring the 590-595nM absorbances of each well on a microplate reader as described above in the methods for the cell proliferation assay.

### Cell migration assay

A non-wounding method using two-well silicone inserts (ibidi; Cat # 80209), each creating a 500 μM gap on cell culture treated 24-well plates, was performed to assess cell migration. Cells were seeded into each of the two wells located within the silicon insert and grown to confluence. When cells reached confluence, the silicone inserts were removed, allowing for cell migration across the 500 μM gap. Time-interval images were captured by phase-contrast microscopy using the BZ-X800 microscopy system (Keyence Corporation of America) to record the progress of cell migration over a 24 hour period and were analyzed by ImageJ software (National Institutes of Health).

### Electric cell-substrate impedance sensing (ECIS) to measure barrier function

Disposable electrode array slides (Applied BioPhysics, 8W10E+) were stabilized with media according to the manufacturer’s protocol. Biological replicates of the generated SLC4A11 V2^WT^, SLC4A11 V3^WT^ and SLC4A11^-/-^ empty hCEnC lines were seeded at 100% confluence in each well of the electrode array and incubated at room temperature for 30 minutes to facilitate a uniform distribution of adherent cells. After seeding and preliminary cell attachment, the electrode arrays were inserted into a 16-well array station that was enclosed within a humidified incubator containing 5% CO_2_ at 37° C. The ECIS Z-theta instrument, connected to the 16 well array station, measured and recorded electric impedance (Ω) at various alternating current (AC) frequencies between 100 Hz and 64,000 Hz for 72 hours. Modeling was performed from electric impedance data using the ECIS software v1.2 (Applied Biophysics) to obtain impedance contributions from cell-cell junctions (*R*_b_, Ω • cm^2^), cell-substrate adhesion (*α*, Ω^1/2^ • cm) and membrane capacitance (C_m_, μF/Cm^2^) [51].

### Immunocytochemistry of cell cultures to determine SLC4A11 subcellular localization

CEnC cultures were grown on glass coverslips and then fixed with 4% paraformaldehyde solution in PBS. Afterwards, the fixed cells were permeabilized in 0.25% Triton X-100 or methanol, and then blocked with PBSCM-T (PBS, 1mM CaCl_2_, 1mM MgCl_2_, and 0.05% Tween20) containing 1% BSA and 10% NGS. For primary CEnC, SLC4A11 protein was detected with a polyclonal rabbit anti-SLC4A11 antibody (PA5-101889, Invitrogen) and custom polyclonal rabbit anti-SLC4A11 antibodies generously provided by Dr. Ira Kurtz [33, 49, 50]. For the generated CEnC lines, SLC4A11 detection was performed using anti-SLC4A11 antibody (PA5-101889, Invitrogen) and custom anti-SLC4A11 antibodies (α-SLC4A11-M36 and α-SLC4A11-V3, generously provided by and characterized by Dr. Joseph Casey’s lab [45]. SLC4A11 antibodies were diluted 1:400 in blocking solution. Mitochondrial protein COX4 was detected with a mouse anti-COX4 antibody (sc-376731, Santa Cruz Biotechnology) diluted 1:200 in blocking buffer. Primary antibody incubation was performed overnight at 4°C. Afterwards, cells were washed with PBSCM-T three times and then incubated with anti-rabbit Alexa Fluor 594 secondary antibody (Thermo Fisher Scientific) diluted 1:500 in blocking buffer for 1–2 hours at room temperature. After the cells were washed again with PBSCM-T three times, they were subsequently mounted with aqueous mounting medium (Vectashield H1200; Vector Laboratories) containing DAPI (4’,6-diamidino-2-phenylindole), and then imaged by fluorescence microscopy.

### Capillary-based western blot to quantify SLC4A11 protein expression

Cells were lysed with RIPA buffer and total protein concentrations of whole-cell lysates were measured by using a Pierce BCA Protein Assay kit (Thermo Fisher Scientific). Protein separation was performed using the Wes separation 12–230 kDa capillary cartridges (Protein Simple) per the manufacturer’s instructions, and detection was achieved with a 1:25 dilution of a custom rabbit anti-SLC4A11 antibody (provided by Dr. Ira Kurtz’s lab at UCLA) and a 1:125 dilution of a mouse GAPDH antibody (MAB374, Millipore). Quantification and data analysis were performed using the Compass for SW software (version 5.0.1).

### Single cell patch-clamp recordings

Single cell recordings were made from adhered CEnC (SLC4A11^-/-^ empty, SLC4A11 V2^WT^ and V3^WT^ hCEnC lines) after seeding cells on 25 mm glass coverslips to ∼2–5% confluence, which were placed into quick change chambers (RC-40LP, Warner Instruments). Membrane voltages were measured using whole-cell patch electrodes in current clamp mode (I_hold_ = 0) using an electrode internal solution consisting of: 125mM K-aspartate, 10mM KCl, 10 mM HEPES, 5 mM NMG-HEDTA, 0.5 mM CaCl2, 0.5 mM MgCl2, 1 mM ATP-Mg, 0.2 mM GTP-Tris, 2.5 mM NADPH; pH was adjusted to ~7.3 with NMG-OH and osmolarity was adjusted to ~280 mOsm. Cells were superfused with bicarbonate-buffered Ames’ media (equilibrated with 5% CO_2_/95% O_2_, pH~7.4), heated to 37°C to measure the baseline membrane voltages of the cells, superfused for 3 minutes with bicarbonate-buffered Ames’ media containing 10mM of NH_4_Cl, then followed by a final wash with NH_4_Cl-free bicarbonate-buffered Ames’ media to remove NH_4_Cl. NH_4_Cl-evoked responses were sampled at 1kHz and digitally low-pass filtered at 50Hz using a 7-pole Butterworth filter. Membrane voltages were averaged during stable periods of baseline and NH_4_Cl treatment.

## Results

*SLC4A11* variants 2 and 3 have been demonstrated to be the only two *SLC4A11* transcript variants expressed in human corneas [45]. In an effort to resolve whether or not the SLC4A11 isoforms encoded by variants 2 and 3 demonstrate distinct functional phenotypes and/or subcellular localization in CEnC, stable hCEnC lines expressing only either wild type *SLC4A11* variant 2 (SLC4A11 V2^WT^ hCEnC), or variant 3 (SLC4A11 V3^WT^ hCEnC) were generated and compared to CEnC in which *SLC4A11* was disrupted by CRISPR-Cas9 mediated gene editing and transduced with lentivirus carrying an empty vector (SLC4A11^-/-^ empty hCEnC). To characterize the impact of CHED- and FECD4-associated *SLC4A11* mutations on CEnC function and SLC4A11 localization, stable SLC4A11 V2^MU^ and V3^MU^ hCEnC lines, each harboring a CHED- or FECD4-associated *SLC4A11* mutation, were also created and compared to the generated SLC4A11 V2^WT^, SLC4A11 V3^WT^ and SLC4A11^-/-^ empty hCEnC lines. Confirmation of the expression of wild type or mutant SLC4A11 protein in each of the generated hCEnC clones was performed using capillary-based immunoblotting (S2 Fig). Each of the SLC4A11 empty^-/-^ hCEnC subclones exhibited no detectable levels of SLC4A11 expression, as expected. SLC4A11 protein expression was detected in each of the SLC4A11 V2^WT^, SLC4A11 V3^WT^, SLC4A11 V2^MU^ and V3^MU^ hCEnC lines, with the exception of SLC4A11 V2^MU Arg125His^ and SLC4A11 V3^MU Arg589X^ hCEnC, which did not express detectable levels of SLC4A11 protein by capillary-based immunoblotting (S2 Fig) and both of which harbor *SLC4A11* nonsense mutations that are suspected to lead to nonsense mediated decay.

### Cell viability in CEnC expressing wild type and mutant *SLC4A11* variant 2 and 3

**Fig 1:**
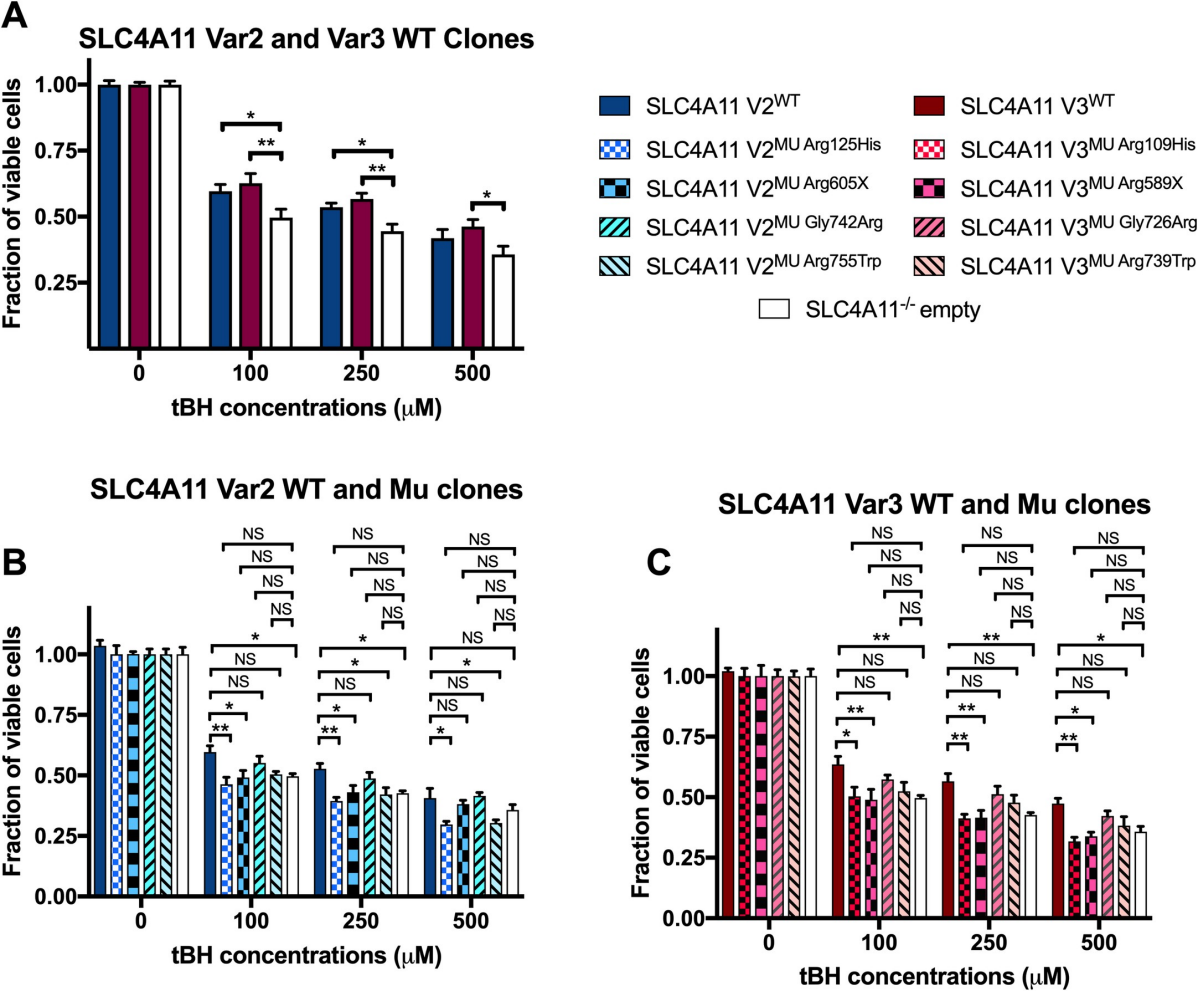
Loss of *SLC4A11* expression and CHED-associated *SLC4A11* mutations in hCEnC lead to decreased cell viability during tBH-induced oxidative stress. (A) Bar graph shows the fraction of viable cells from SLC4A11 V2^WT^, SLC4A11 V3^WT^, and SLC4A11^-/-^ empty hCEnC lines after induction of acute oxidative stress by various concentrations of tBH. Within each hCEnC line, individual measurements were normalized to the levels of total viable non-treated cells that were seeded and cultured in parallel. Bar graphs show the normalized fraction of viable cells from (B) SLC4A11 V2^MU^ hCEnC lines and (C) SLC4A11 V3^MU^ hCEnC lines after induction of acute oxidative stress by various concentrations of tBH. Data in bar graphs are represented as mean ±SEM (n = 8). Statistical comparisons were performed using two-way ANOVA with Tukey’s multiple comparison test. *, P<0.05; **, P<0.01; NS, not significant.

A previous report has shown knockdown of *SLC4A11* leads to decreased NRF2-mediated antioxidant response in CEnC and that transient overexpression of *SLC4A11* increased CEnC viability in response to acute oxidative stress induced with tBH exposure [43]. However, it is unclear whether all expressed transcript variants of *SLC4A11*, or just a subset of *SLC4A11* variants, confer oxidative stress protection in CEnC. Therefore, to determine whether *SLC4A11* variant 2 and/or variant 3 plays a role in CEnC viability during oxidative stress, our generated stable SLC4A11 V2^WT^, SLC4A11 V3^WT^, and SLC4A11^-/-^ empty hCEnC lines were subjected to acute oxidative stress induction with various concentrations of tBH for 2.5 hours. MTT cell viability assays were performed to assess the proportion of viable cells during tBH treatment, which were normalized to the total viable cells that were not treated and cultured in parallel. Compared to SLC4A11^-/-^ empty hCEnC, both SLC4A11 V2^WT^ hCEnC and SLC4A11 V3^WT^ hCEnC retained significantly higher levels of viable cells under acute oxidative stress induced with 100 μM and 250 μM of tBH induction, while SLC4A11 V3^WT^ hCEnC also retained significantly higher levels of cell viability with 500 μM of tBH induction (Fig 1A).

To elucidate the impact of CHED- and FECD4-associated *SLC4A11* mutations on CEnC viability during oxidative stress, cell viability assays were also performed on SLC4A11 V2^MU^ and V3^MU^ hCEnC lines that were exposed to an acute oxidative stress induced by tBH treatment. None of the SLC4A11 mutant hCEnC lines exhibited a significant increase in cell viability following tBH treatment when compared to SLC4A11^-/-^ empty hCEnC. When compared to SLC4A11 V2^WT^ and V3 ^WT^ hCEnC, CHED-associated SLC4A11 V2^MU Arg125His^ (and the equivalent variant 3-specific mutation SLC4A11 V3^MU Arg109His^) and SLC4A11 V2^MU Arg605X^ (and the equivalent variant 3-specific mutation SLC4A11 V3^MU Arg589X^) hCEnC lines demonstrated significantly decreased cell viability following exposure to 100 μM, 250 μM and 500 μM tBH, with the exception of a non-significant decrease of SLC4A11 V2^MU Arg605X^ following exposure to 500 μM tBH (Fig 1B and 1C).

### Cell proliferation in CEnC expressing wild type and mutant *SLC4A11* variant 2 and 3

**Fig 2:**
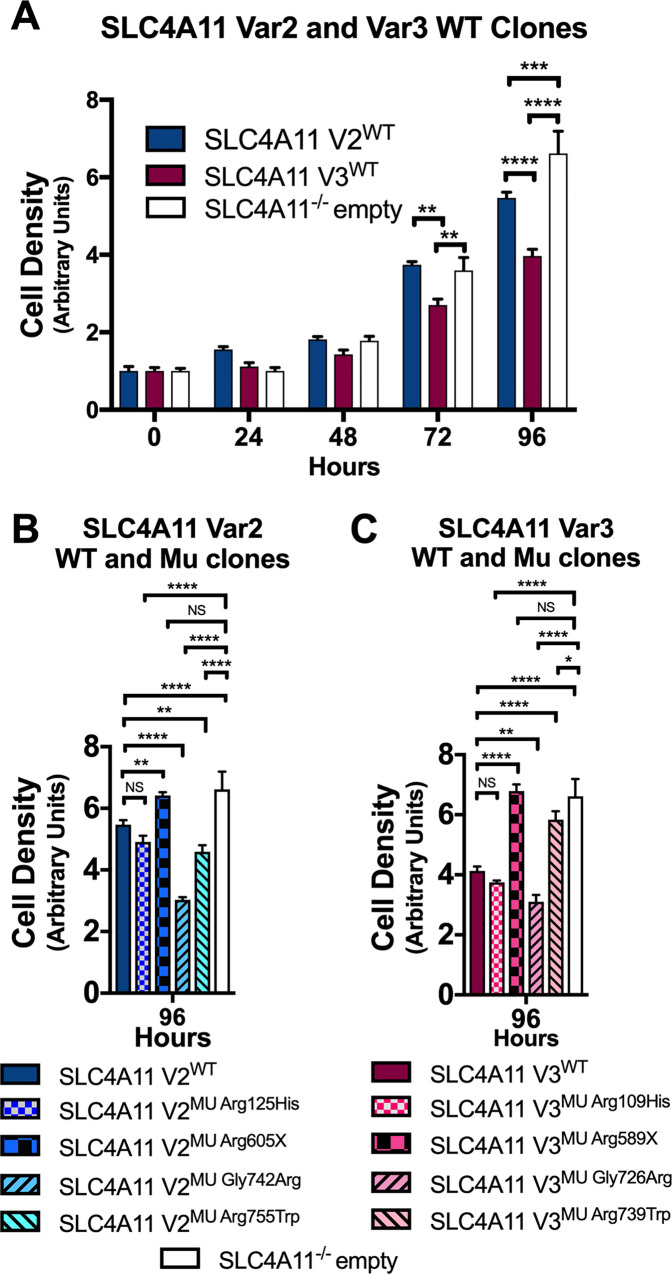
Loss of *SLC4A11* expression and CHED- and FECD4-associated *SLC4A11* mutations in hCEnC alter CEnC proliferation rates. (A) Bar graph shows the relative cell densities of SLC4A11 V2^WT^, SLC4A11 V3^WT^, and SLC4A11^-/-^ empty hCEnC lines that were initially seeded in parallel at ~2000 cells/well. Cell densities were measured using MTT assays over a 96 hour time-course and then normalized to the initial cell densities of each hCEnC line. Bar graphs show the normalized cell densities of (B) SLC4A11 V2^MU^ hCEnC lines and (C) SLC4A11 V3^MU^ hCEnC lines. Data in bar graphs are represented as mean ±SEM (n = 4). Statistical comparisons were performed using two-way ANOVA with Tukey’s multiple comparison test. *, P<0.05; **, P<0.01; ***, P<0.001; ****, P<0.0001; NS, not significant.

In vivo, hCEnC are arrested in the G1 phase of the cell cycle, resulting in a gradual CEnC loss of 0.3 to 0.6% per year in healthy adults although the endothelial cell reserve is sufficient to maintain corneal clarity [52]. In contrast, individuals affected with CHED have insufficient CEnC densities to maintain corneal clarity at birth or in early childhood. To determine whether or not *SLC4A11* variants 2 and/or 3 have roles in regulating CEnC proliferation, SLC4A11 V2^WT^ and V3^WT^ hCEnC were seeded at low density (2000 cells per well) in 96 well culture plates and cell proliferation rates were monitored by measuring cell density over a 96 hour time course. SLC4A11^-/-^ empty hCEnC exhibited significantly higher cell densities compared to SLC4A11 V2^WT^ at 96 hours post-seeding and SLC4A11 V3^WT^ hCEnC at 72 and 96 hours post-seeding (Fig 2A).

The cell densities of the SLC4A11 V2^MU^ and V3^MU^ hCEnC lines were measured over a 96 hour time course to characterize the impact of CHED- and FECD4-associated SLC4A11 mutations on CEnC proliferation. By 96 hours post-seeding, CHED-associated SLC4A11 V2^MU Arg755Trp^ and FECD4-associated SLC4A11 V2^MU Gly742Arg^ (and the equivalent variant 3-specific mutation SLC4A11 V3^MU Gly726Arg^) hCEnC demonstrated significantly lower cell densities compared to their respective SLC4A11 wild type hCEnC lines (Fig 2B and 2C). In contrast, CHED-associated SLC4A11 V2^MU Arg605X^ (and the equivalent variant 3-specific mutation SLC4A11 V3^MU Arg589X^) and SLC4A11 V3^MU Arg739Trp^ mutations exhibited increased cell densities compared to their respective SLC4A11 wild type hCEnC lines.

### Cell migration in CEnC expressing wild type and mutant *SLC4A11* variant 2 and 3

**Fig 3:**
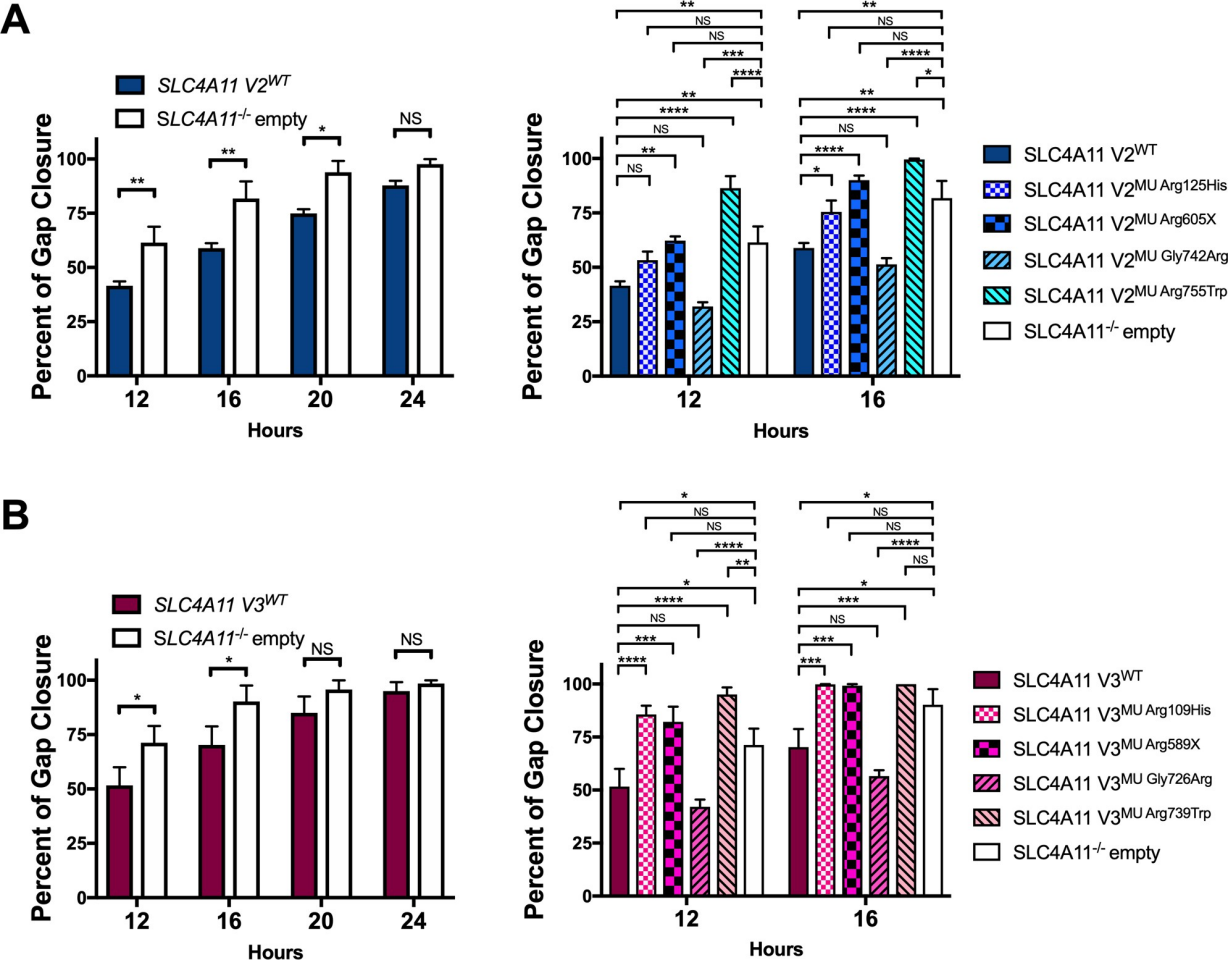
Loss of *SLC4A11* expression and CHED-associated *SLC4A11* mutations in hCEnC lead to increased hCEnC migration rates. Bar graphs show the percentages of gap closure of a non-wounding 500μM gap between confluent cells at various time intervals by migrating (A) SLC4A11 V2^WT^ and SLC4A11 V2^MU^ hCEnC lines, and (B) SLC4A11 V3^WT^ and SLC4A11 V3^MU^ hCEnC lines. Data are represented as mean ±SEM (n≥3). Statistical comparisons were performed using two-way ANOVA with Tukey’s multiple comparison test. *, P<0.05; **, P<0.01; ***, P<0.001; ****, P<0.0001; NS, not significant.

During CEnC loss, the corneal endothelium utilizes cell migration as a compensatory mechanism to preserve the function and integrity of the corneal endothelium, which involves the spreading and migrating of neighboring CEnC to cover the areas devoid of cells [53]. To elucidate whether or not *SLC4A11* variants 2 and/or 3 have roles in CEnC migration, SLC4A11 V2^WT^ hCEnC, SLC4A11 V3^WT^ hCEnC and SLC4A11^-/-^ empty hCEnC were seeded and grown to confluence within adjacent pairs of wells divided by a silicone insert, which when removed, created a non-wounding 500 μM gap between confluent monolayers of seeded cells from each adjacent pair of wells. The closure of the 500 μM gap by migrating CEnC was monitored by phase-contrast imaging over a time-course of 24 hours or more after removal of the insert. Compared to SLC4A11^-/-^ empty hCEnC, SLC4A11 V2^WT^ and V3^WT^ hCEnC exhibited significantly lower gap closure percentages (i.e. lower migration rates) at 12 and 16 hours with no significant difference at 24 hours (Fig 3A and 3B).

The cell migration rates of SLC4A11 V2^MU^ and V3^MU^ hCEnC lines were also assessed by the non-wounding two-well silicone insert method. At 12 and 16 hours after removing the insert, CHED-associated SLC4A11 V2^MU Arg125His^ (and the equivalent variant 3-specific mutation SLC4A11 V3^MU Arg109His^), SLC4A11 V2^MU Arg605X^ (and the equivalent variant 3-specific mutation SLC4A11 V3^MU Arg589X^) and SLC4A11 V2^MU Arg755Trp^ (and the equivalent variant 3-specific mutation SLC4A11 V3^MU Arg739Trp^) hCEnC demonstrated increased migration rates compared with their respective SLC4A11 wild type hCEnC lines, with the exception of a non-significant increase in the rate of SLC4A11 V2^MU Arg125His^ hCEnC at 12 hours (Fig 3A and 3B). In contrast, the migration rates for FECD4-associated SLC4A11 V2^MU Gly742Arg^ (and the equivalent variant 3-specific mutation SLC4A11 V3^MU Gly726Arg^) hCEnC were not significantly different from their respective SLC4A11 wild type hCEnC lines.

### Characterization of cell barrier function in CEnC expressing wild type and empty *SLC4A11* variants 2 and 3

**Fig 4:**
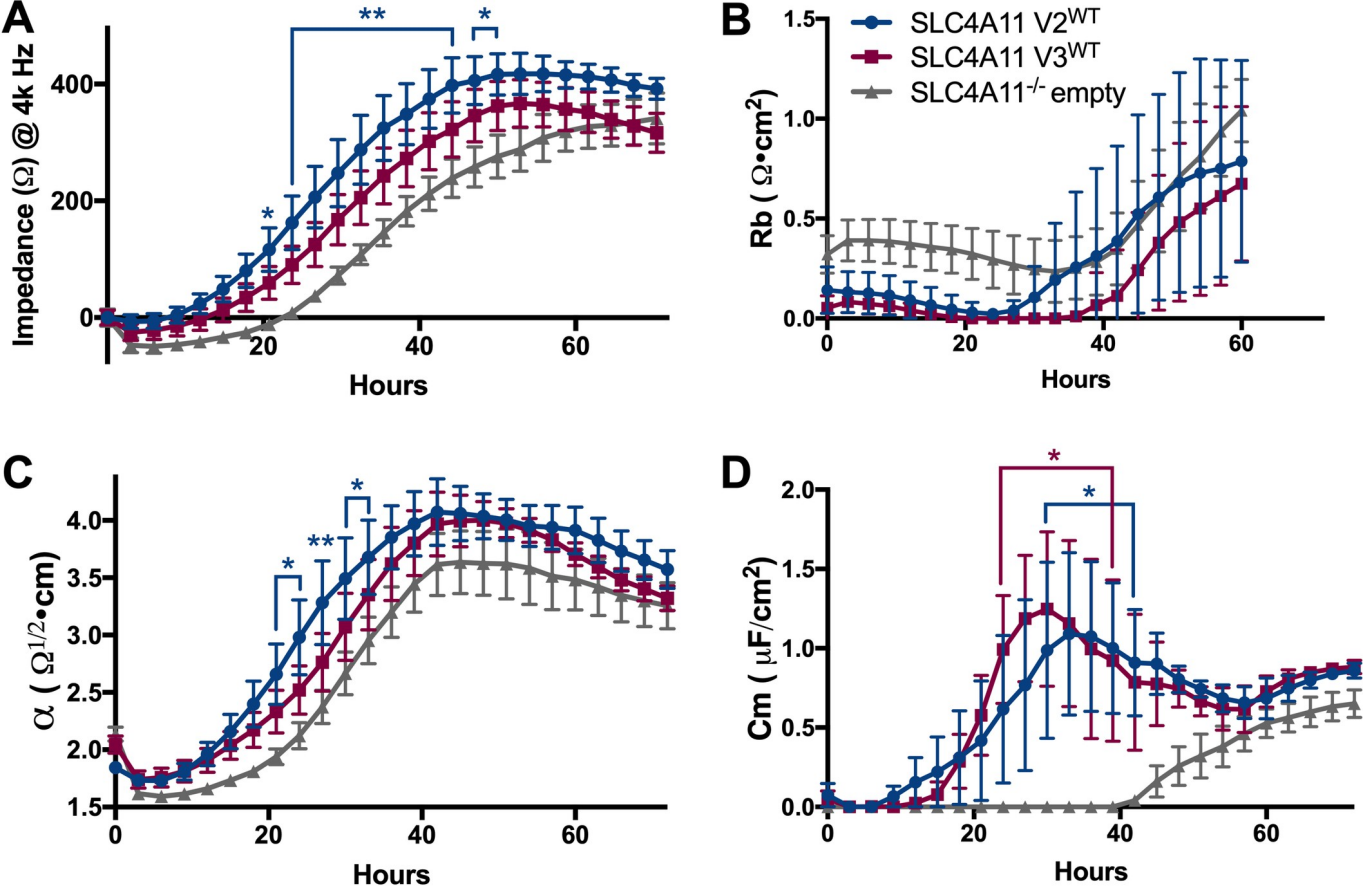
Loss of *SLC4A11* expression in hCEnC leads to decreased cell barrier function. Line graphs show the (A) impedances (ohms) at 4000 Hz; (B) impedance contributions from cell-cell junctions (*R*_b_, Ω • cm^2^); (C) cell-substrate adhesion (*α*, Ω^1/2^ • cm); and (D) membrane capacitance (C_m_, μF/Cm^2^) of SLC4A11 V2^WT^, SLC4A11 V3^WT^, and SLC4A11^-/-^ empty hCEnC lines that were seeded at 100% confluence into a multi-well electrode array and measured for electric impedances at various frequencies (Hz) over a ~72 hour time-course. Data in bar graphs are represented as mean ±SEM (n≥4). Statistical comparisons between SLC4A11 V2^WT^ and SLC4A11^-/-^ empty and between SLC4A11 V3^WT^ and SLC4A11^-/-^ empty were performed using two-way ANOVA with Dunnett’s multiple comparison test at each time point. Time points shown with asterisks denote significant differences between SLC4A11^WT^ (V2^WT^ or V3^WT^) and SLC4A11 empty^-/-^ hCEnC. *, P<0.05; **, P<0.01; NS, not significant.

SLC4A11 is reported to serve as a cell adhesion molecule in CEnC, which bind to the extracellular matrix proteins on the Descemet membrane [41]. Cell adhesion is a critical component of cell barrier function, which is necessary for semi-permeable tissue such as the corneal endothelium that regulates the transport of ions and osmotically driven water flux to maintain corneal clarity. To assess the role of *SLC4A11* variants 2 and 3 on cell barrier function, electric cell-substrate impedance sensing (ECIS) was performed on SLC4A11 V2^WT^ and V3^WT^ hCEnC, and SLC4A11^-/-^ empty hCEnC, which were each grown to confluence on an electrode array and then measured for electric impedance (Ω) at multiple alternating current frequencies (Hz) over a time-course. While both SLC4A11 V2^WT^ and V3^WT^ hCEnC exhibited overall increases in impedances compared to SLC4A11^-/-^ empty hCEnC at a frequency of 4000 Hz, only the impedances demonstrated by SLC4A11 V2^WT^ hCEnC (between ~21 and ~53 hours post-seeding) were significantly higher compared to SLC4A11^-/-^ empty hCEnC (Fig 4A).

Modeling was performed on impedance data, acquired from a range of frequencies between 100 Hz and 64,000 Hz, to evaluate the impedance contributions from cell-cell junctions (*R*_b_, Ω • cm^2^), cell-substrate adhesion (*α*, Ω^1/2^ • cm), and membrane capacitance (C_m_, μF/Cm^2^) of each of the hCEnC lines. SLC4A11^-/-^ empty hCEnC not did exhibit any significant differences in impedances at cell-cell junctions compared to SLC4A11 V2^WT^ or V3^WT^ hCEnC (Fig 4B). However, SLC4A11^-/-^ empty hCEnC demonstrated decreased impedances in cell-substrate adhesion and membrane capacitance compared to both SLC4A11 V2^WT^ and V3^WT^ hCEnC (Fig 4C and 4D).

### Characterization of cell membrane voltage potential in CEnC expressing wild type, mutant and knockout *SLC4A11* variants 2 and 3 under ammonium induction

**Fig 5:**
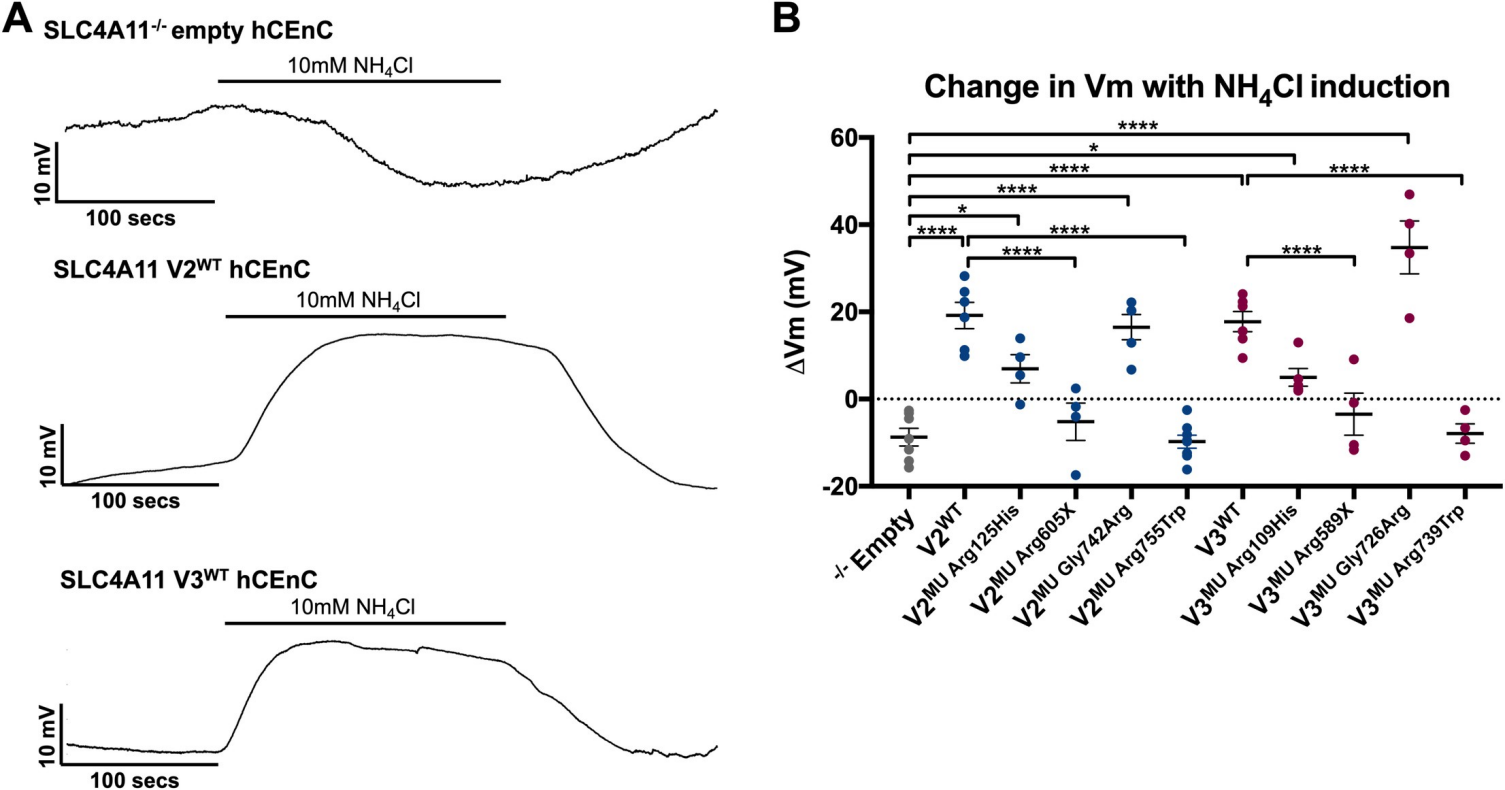
Loss of *SLC4A11* expression and *SLC4A11* mutations lead to altered membrane potential responses in single-cell current-clamp recordings in hCEnC. (A) Representative single-cell current-clamp responses of SLC4A11^-/-^ empty hCEnC, SLC4A11 V2^WT^ and V3^WT^ hCEnC when induced with 10mM NH_4_Cl. (B) Scatterplot depicts the change in membrane voltage (Vm) measured by single-cell current-clamp recordings of SLC4A11^-/-^ empty, SLC4A11 V2^WT^, SLC4A11 V3^WT^, SLC4A11 V2^MU^ and SLC4A11 V3^MU^ hCEnC lines during induction with 10mM NH_4_Cl. ΔVm for each cell line was analyzed (n≥4) and statistical comparisons were performed using one-way ANOVA with Tukey’s multiple comparison test. Mean ±SEM; *, P<0.05; **, P<0.01; ***, P<0.001; ****, P<0.0001.

SLC4A11 is characterized as an electrogenic membrane transporter that is sensitive to NH_4_Cl, and is hypothesized to function either as a co-transporter of NH_3_:H^+^ or as a NH_3_-activated H^+^ transporter [31]. Single cell voltage clamp recordings were performed to measure the responses in membrane potential of SLC4A11^-/-^ empty, SLC4A11 V2^WT^ and V3^WT^ hCEnC lines that were extracellularly perfused with 10mN NH_4_Cl (Fig 5A). Exposure to NH_4_Cl induced a -8.717 mV (SEM ± 2.029 mV) hyperpolarization in SLC4A11^-/-^ empty hCEnC. In contrast, the membranes of SLC4A11 V2^WT^ and V3^WT^ hCEnC depolarized by +19.19 mV (SEM ± 2.998 mV) and +17.76 mV (SEM ± 2.326 mV), respectively, when exposed to NH_4_Cl.

Single cell recordings were also performed in the SLC4A11 V2^MU^ and V3^MU^ hCEnC lines (Fig 5). Similar to their SLC4A11^WT^ counterparts, SLC4A11 V2^MU Gly742Arg^ (+16.5 mV, SEM ± 2.903 mV) and SLC4A11 V3^MU Gly726Arg^ (+34.78 mV, SEM ± 6.064 mV) demonstrated membrane depolarization when induced with NH_4_Cl. While NH_4_Cl-induced depolarization of SLC4A11 V2^MU Arg125His^ (+6.942 mV, SEM ± 3.231 mV) and SLC4A11 V3^MU Arg109His^ (+4.982 mV, SEM ± 2.052 mV) hCEnC were also observed, the intensities of depolarization were significantly diminished compared to their respective SLC4A11 wild type counterparts. Similar to SLC4A11^-/-^ empty hCEnC, NH_4_Cl-induced hyperpolarization was observed in SLC4A11 V2^MU Arg605X^ (-5.194 mV, SEM ± 2.903 mV) (and the equivalent variant 3-specific mutation SLC4A11 V3^MU Arg589X^ (-3.466 mV, SEM ± 4.835 mV)) and SLC4A11 V2^MU Arg755Trp^ (-9.754 mV, SEM ± 1.487 mV) (and the equivalent variant 3-specific mutation SLC4A11 V3^MU Arg739Trp^ (-7.883 mV, SEM ± 2.221 mV)) hCEnC.

### SLC4A11 localization in primary HCEnC

**Fig 6:**
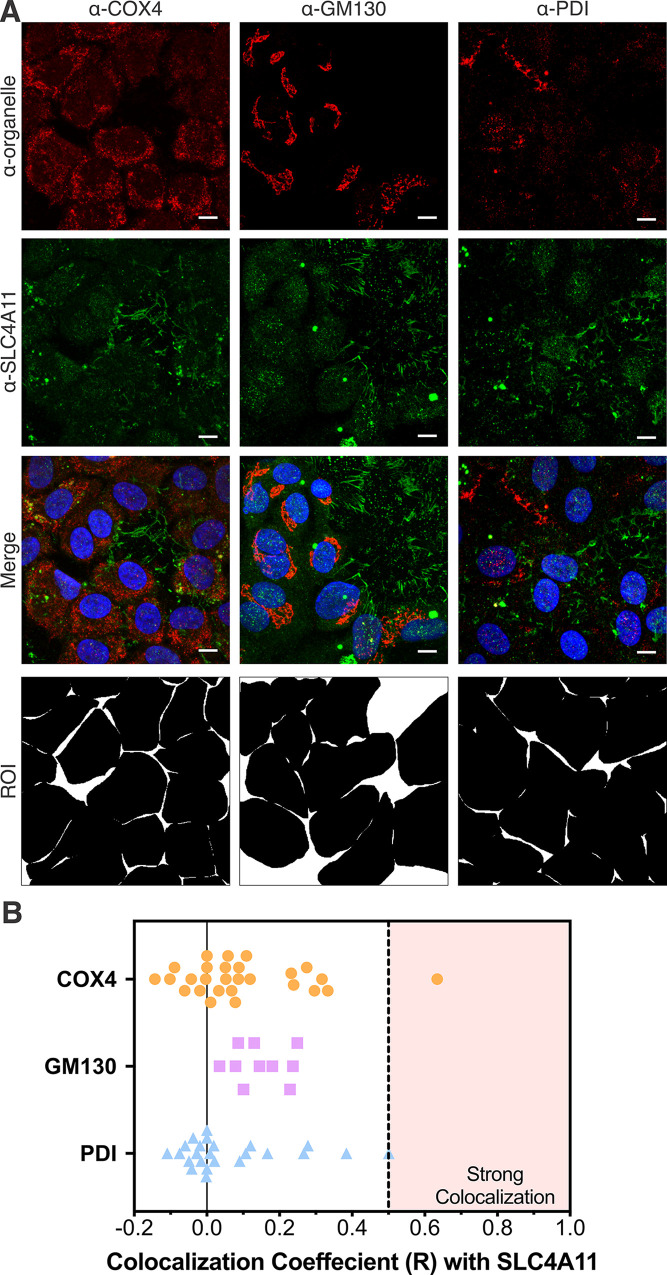
SLC4A11 localizes to the plasma membrane and mitochondrial punctate structures in primary hCEnC. (A) Confocal fluorescence microscopy images of primary hCEnC stained with anti-organelle antibodies (red, anti-COX4, anti-GM130 and anti-PDI, respectively) and an anti-SLC4A11 antibody (green, custom-made). Region of interests (ROI) filters used for computing SLC4A11 colocalization coefficient with organelle markers at single-cell level are shown in bottom row. Scale bars: 10 μm. (B) Summary plot of the distribution of colocalization coefficient (R) of SLC4A11 with organelle makers. Range of R values considered as “strong colocalization” is shaded.

The subcellular localization of SLC4A11 to the plasma membrane has been well-characterized. However, in a more recent report, SLC4A11 was demonstrated to also localize to the inner- mitochondrial membranes [38]. To obtain more clarity regarding the subcellular localization of SLC4A11, immunocytochemistry and fluorescence microscopy of primary hCEnC were performed using various anti-SLC4A11 antibodies. Immunostaining of SLC4A11 in primary hCEnC with a custom-made affinity purified polyclonal rabbit anti-SLC4A11 antibody [54], recognizing all of the three major SLC4A11 isoforms, demonstrated predominately basolateral plasma membrane localization of SLC4A11 (Fig 6A). Co-staining with antibody recognizing mitochondrial COX4 revealed SLC4A11 partially colocalizing with COX4 within punctate globular intracellular structures in a few cells but not with the majority of COX4 stained mitochondria (Fig 6A and 6B). Additionally, minimal colocalization was noted with antibodies recognizing organelle markers GM130 (Golgi) and PDI (endoplasmic reticulum; ER) (Fig 6A and 6B). Immunostaining of SLC4A11 in primary hCEnC with a polyclonal rabbit anti-SLC4A11 antibody from Invitrogen (PA5-101889) that recognizes all three major SLC4A11 isoforms demonstrated plasma membrane and intracellular localization of SLC4A11 (S3 Fig). Co-staining with an anti-COX4 antibody revealed partial colocalization of SLC4A11 and COX4 within punctate globular intracellular structures but absence of SLC4A11 in mitochondrial filamentous structures that COX4 independently stained (S3 Fig).

To determine whether the SLC4A11 protein isoforms encoded by variants 2 and 3 localize to different subcellular regions, immunostaining of primary hCEnC and fluorescence microscopy were performed with custom anti-SLC4A11 antibodies (generously provided by Dr. Joseph Casey’s lab): α-SLC4A11-M36 (recognizing each of the major SLC4A11 isoforms) and α-SLC4A11-V3 (recognizing SLC4A11 isoform C encoded by variant 3) [45]. Similar to the staining by the Invitrogen anti-SLC4A11 antibody, the α-SLC4A11-M36 antibody primarily stained the plasma membrane (S4A and S4B Fig). While punctate structures within a subset of primary CEnC were partially co-stained with the α-SLC4A11-M36, α-SLC4A11-V3 and anti-COX4 antibodies, structures that were presumably mitochondria were independently stained with the anti-COX4 antibody but not the α-SLC4A11-M36 or α-SLC4A11-V3 antibodies (S4 Fig).

### SLC4A11 localization in wild type and mutant *SLC4A11* variant 2 and 3 hCEnC

**Fig 7:**
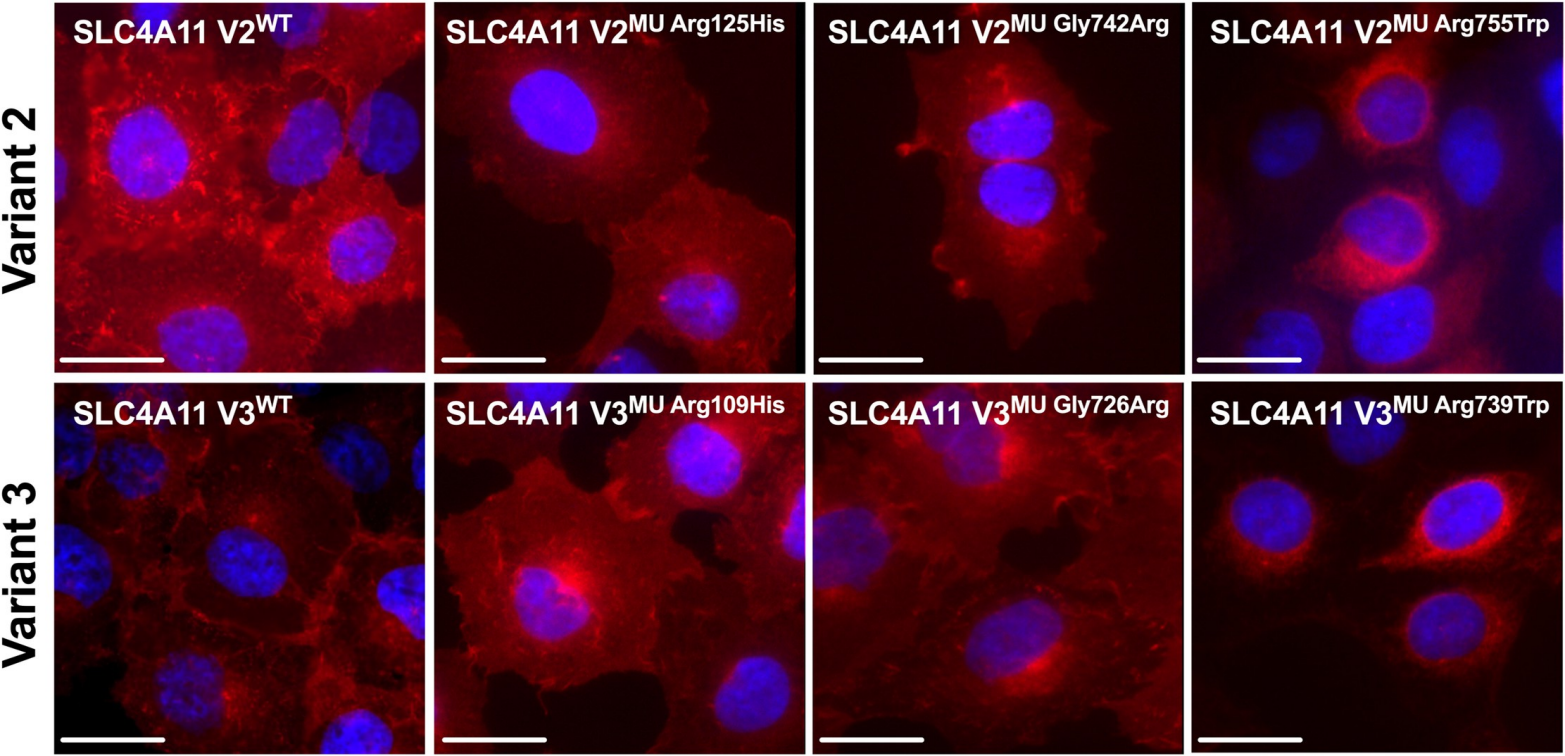
SLC4A11 variants 2 and 3 predominantly localize to the plasma membrane in hCEnC expressing either wild type or CHED-/FECD4-associated *SLC4A11* mutations. Fluorescence microscopy images of SLC4A11 V2^WT^ and V2^MU^ hCEnC (top row), SLC4A11 V3^WT^ and V3^MU^ hCEnC (bottom row) stained with an anti-SLC4A11 antibody (Invitrogen) (red) and DAPI (blue). Scale bars: 20 μm.

Immunostaining of SLC4A11 V2^WT^ and V3^WT^ hCEnC with Invitrogen’s anti-SLC4A11 antibody, the α-SLC4A11-M36 antibody and the α-SLC4A11-V3 antibody demonstrated SLC4A11 isoforms B and C predominately localizing to the plasma membrane (Fig 7 and S5 Fig). Immunostaining of SLC4A11 V2^MU^ and V3^MU^ hCEnC demonstrated SLC4A11 localization primarily to the cell surface in SLC4A11 V2^MU Arg125His^ and SLC4A11 V3^MU Arg109His^, as well as SLC4A11 V2^MU Gly742Arg^ and SLC4A11 V3^MU Gly726Arg^ hCEnC, similar to their wild type counterparts (Fig 7). In contrast, perinuclear staining of SLC4A11 was observed in SLC4A11 V2^MU Arg755Trp^ (and the equivalent variant 3-specific mutation SLC4A11 V3^MU Arg739Trp^) hCEnC, while little to no SLC4A11 expression was observed in SLC4A11 V2^MU Arg605X^ (or the equivalent variant 3-specific mutation SLC4A11 V3^MU Arg589X^) hCEnC, both of which are predicted to cause a premature stop codon in SLC4A11 (Fig 7).

## Discussion

The primary function of the corneal endothelial cells is to maintain the cornea in a slightly dehydrated state, which is essential for corneal clarity. While studies have identified several CEnC functions that are facilitated by SLC4A11 (e.g. ion transport and cell adhesion), the role of SLC4A11 in maintaining the density of CEnC is unclear. While individuals affected with FECD typically exhibit a progressive decrease in CEnC density by the fifth decade of life, it is unclear whether infants and young children with CHED have decreased CEnC densities, and if so, whether the decrease in CEnC density is due to the loss of the proliferative capacity of CEnC during development and/or a decrease in CEnC viability. The finding that *Slc4a11* knockout mice have approximately the same CEnC density as wild type mice at 10 weeks of age, but exhibit a significantly lower CEnC density by 40 weeks of age, suggests that decreased CEnC viability may be the cause of the congenital corneal edema in individuals with CHED [55].

We hypothesize that a decrease in CEnC viability secondary to oxidative stress leads to the loss of CEnC density and contributes to the pathogenesis of both FECD and CHED. Herein, we demonstrate that loss of SLC4A11 in HCEnC results in decreased cell viability under tBH-induced oxidative stress compared with cells stably transduced with SLC4A11, providing evidence that SLC4A11 offers oxidative stress protection. These findings corroborate previous studies that demonstrated *SLC4A11* variant 2 knockdown leads to decreased primary hCEnC viability, and that variant 3 (in addition to variant 2) functions to provide oxidative stress protection in CEnC (Fig 1A) [43]. Additionally, we show the selected CHED- and FECD4-associated *SLC4A11* mutations in this study did not confer significant increases in cell viability when compared to the SLC4A11^-/-^ empty CEnC, which suggest these *SLC4A11* mutations are not protective against oxidative stress.

We next sought to determine whether CHED- and FECD4-associated *SLC4A11* mutations could influence endothelial cell density through mechanisms other than cell viability, such as cell proliferation, migration and adhesion. While CEnC can be induced to proliferate in culture, mature post-mitotic CEnC in vivo are arrested in the G1 phase of the cell cycle and typically only migrate, and enlarge, when neighboring CEnC are lost due to trauma or disease [56]. Given the dynamic between CEnC proliferation, migration, and cell adhesion [57, 58], we examined the role of SLC4A11 in each of these three functions and discovered SLC4A11^-/-^ empty hCEnC exhibited increased proliferation and migration rates and demonstrated decreased levels of cell-substrate adhesion compared to hCEnC transduced with either *SLC4A11* variant 2 or variant 3. The observed decreased levels of cell-substrate adhesion associated with the loss of SLC4A11 in hCEnC corroborates a previous study implicating that SLC4A11 serves as a cell adhesion molecule that directly binds to extracellular matrix proteins [41]. However, the mechanism by which the loss of SLC4A11 expression in CEnC leads to increased endothelial cell proliferation and migration is unclear. Further investigation will aid in determining whether the role of SLC4A11 in CEnC adhesion is to modulate cell proliferation and migration, or whether novel SLC4A11-dependent signaling pathways could explain the changes in proliferation and migration associated with *SLC4A11* mutations.

As the localization of SLC4A11 to the hCEnC surface is consistent with a role for SLC4A11 in hCEnC proliferation, migration and adhesion, a purported role in protection from oxidative stress would suggest mitochondrial localization as well. Indeed, SLC4A11 has been shown to target inner-mitochondrial membranes, in spite of in silico proteomic analyses predicting each of the three isoforms of SLC4A11 to have a low probability of mitochondrial targeting based on the absence of canonical N-terminal mitochondrial targeting sequences. The lack of a canonical mitochondrial targeting sequence in SLC4A11 suggests that SLC4A11 traffics to the mitochondria via chaperone-mediated carrier pathways that recognize cryptic internal mitochondrial targeting signals instead [38, 59]. Supporting this hypothesis, LC-MS/MS analyses identified mitochondrial chaperone proteins HSP90 and HSC70 bound to HA-tagged SLC4A11 isoform B (encoded by variant 2) in SLC4A11-transfected PS120 fibroblast cell and mitochondrial lysates, and inactivation of these chaperones was shown to disrupt mitochondrial SLC4A11 translocation [59]. We investigated whether the SLC4A11 isoforms encoded by variants 2 and 3 exhibit differential trafficking to the cell surface and/or mitochondria in hCEnC. For both variant 2 and variant 3, the majority of SLC4A11 was observed at the cell surface with ICC in our variant-specific hCEnC lines, and only partial colocalization of SLC4A11 was observed with mitochondrial marker COX4 in primary hCEnC. Interestingly, this partial colocalization between SLC4A11 and COX4 was confined to punctate structures that reside adjacent to the network of filamentous mitochondrial structures. Mitochondrial morphology consists of four major classifications: network, unbranched, swollen, and punctate, with the primary morphology of mitochondria undergoing mitophagy classified as punctate [60]. Thus, based on mitochondrial morphology, it is possible that the role of these COX4/SLC4A11 co-expressing mitochondrial puncta involves mitophagy, the selective degradation of mitochondria through autophagy. Though additional investigational studies are needed, this proposal is supported by the recent link between SLC4A11 and activation/expression of many molecular components (e.g. BECN1, ULK, LC3) that are involved in autophagy and mitophagy [61, 62].

We performed single-cell recordings to measure the impact of SLC4A11 loss on membrane potential in hCEnC and demonstrated membrane depolarization of SLC4A11 V2^WT^ hCEnC and SLC4A11 V3^WT^ hCEnC, while hCEnC that lack SLC4A11 protein expression exhibited membrane hyperpolarization in response to extracellularly perfused 10mM NH_4_Cl. Interestingly, hCEnC which expressed but mislocalized SLC4A11 to the perinuclear regions, also exhibited membrane hyperpolarization, indicating that SLC4A11 must be at the cell surface in order to properly modulate membrane potential. This work recapitulates our previous study using the same single-cell recording approach in mouse CEnC (mCEnC) that demonstrated membrane depolarization of *Slc4a11*^+/+^ mCEnC and membrane hyperpolarization of *Slc4a11*^-/-^ mCEnC during 10mM NH_4_Cl perfusion, providing further functional evidence that *Slc4a11*/*SLC4A11* likely possess a high degree of gene orthology [35]. In the same study, RNA-sequencing of *Slc4a11*^+/+^ and *Slc4a11*^-/-^ mCEnC identified differential expression of dozens of genes encoding ion channels or transporters, providing evidence for a role of Slc4a11 in modulating ion transport in CEnC. Future investigation could elucidate whether a similar set of differentially expressed ion channels and transporters are associated with the loss of SLC4A11 expression and/or SLC4A11 mislocalization in hCEnC.

In this study, the exogenous expression of wild type *SLC4A11* variants 2 and 3 in hCEnC demonstrated similar cell functional profiles (e.g. decreased CEnC proliferation, decreased migration, increased viability under oxidative stress and increased electric impedances compared to SLC4A11^-/-^ empty hCEnC, as well as membrane depolarization in response to extracellular NH_4_Cl induction). Together, these findings suggest that variants 2 and 3 likely encode protein isoforms that have significantly overlapping roles in CEnC, despite having divergent N-terminal sequences that lead to marked predicted protein structure disparities at the N-terminal as seen in previous structural homology modelling [45]. The functional similarity between variants 2 and 3 in this study is consistent with a previous report documenting no significant difference in H^+^ transport and water flux activity in HEK293 transfected with variant 2 or variant 3 [33, 45].

It is unclear whether or not heterozygous *SLC4A11* mutations found in the parents of individuals affected with CHED are causative for FECD4. Chaurasia et al. evaluated eight individuals with CHED who harbored homozygous *SLC4A11* mutations, and their parents, who were heterozygous the *SLC4A11* mutations, and reported that 10 of the 16 parents demonstrated early signs of FECD [28]. This suggests that heterozygous *SLC4A11* mutations in parents of individuals affected with CHED may be sufficient to cause FECD4 and thus it follows that a higher incidence of FECD would be observed in regions where CHED is more prevalent. However, while FECD is the indication for approximately 4–5% of corneal transplants performed globally, FECD is the indication for only approximately 1–3% of corneal transplants performed in India, where the majority of CHED cases have been reported [63–66]. Therefore, it is not clear whether the zygosity of *SLC4A11* mutations primarily determines an individual will develop CHED or FECD, or whether *SLC4A11* mutations exhibit varying impacts on CEnC function that can lead to: CHED (when homozygous) but not FECD (when heterozygous); FECD (when heterozygous) but not CHED (when homozygous); or both CHED and FECD, depending on the zygosity.

To attempt to answer this question, we compared the impact of three CHED-associated *SLC4A11* mutations and a FECD4-associated *SLC4A11* mutation on hCEnC function. Although FECD4 is associated with heterozygous *SLC4A11* mutations, we stably transduced both CHED- and FECD4-associated *SLC4A11* mutations to mimic a homozygous state, which allows for a comparison of the functional impact of each mutation by controlling for zygosity. Oxidative stress in CEnC is considered to be a major contributor to the pathogenesis of both FECD and CHED [43, 67]. Similar to CEnC harboring CHED-associated *SLC4A11* mutations, CEnC harboring the FECD4-associated mutation c.2224G>A (Gly742Arg) (and variant 3 equivalent c.2176G>A (Gly726Arg)) did not exhibit any significant increases in cell viability during tBH-induced oxidative stress when compared to SLC4A11^-/-^ empty CEnC, suggesting both CHED- and FECD4-associated *SLC4A11* mutations lead to a loss of oxidative stress protection similar to that of the *SLC4A11* empty CEnC. However, unlike most of the CHED-associated *SLC4A11* mutations examined in this study, FECD4-associated mutation c.2224G>A (Gly742Arg) (and variant 3 equivalent c.2176G>A (Gly726Arg)) also did not demonstrate significant changes in CEnC viability under oxidative stress when compared to their wild type counterparts, which means additional studies are needed to parse out the impact of these FECD-associated *SLC4A11* mutations on cell viability. Overall, with the sole exception of CEnC proliferation, FECD4-associated mutation c.2224G>A (Gly742Arg) (and variant 3 equivalent c.2176G>A (Gly742Arg)) did not significantly impact SLC4A11 cell surface localization or CEnC functions that were investigated in this study, while each of the three CHED-associated *SLC4A11* mutations significantly altered two or more CEnC functions. Thus, the *SLCA411* mutation associated with FECD4 has less of an impact *in vitro* on CEnC functional profiles compared to the three *SLC4A11* mutations associated with CHED.

In summary, CHED-associated *SLC4A11* mutations likely lead to CEnC dysfunction, and ultimately CHED, by altering cell migration, proliferation, viability, membrane conductance, barrier function, and/or cell surface localization of the SLC4A11 protein in CEnC. In contrast, the FECD4-associated *SLC4A11* mutation c.2224G>A is functionally distinct from the CHED-associated *SLC4A11* mutations, impacting only hCEnC proliferation. SLC4A11 protein isoforms encoded by *SLC4A11* variants 2 and 3 both primarily localize to the hCEnC surface, with subcellular targeting to mitochondrial punctate structures, and likely have highly overlapping functional roles in CEnC. Investigations of the functional impact of additional CHED-associated and FECD4-associated *SLC4A11* mutations on CEnC function will determine whether the difference in the phenotype of the two dystrophies is primarily dependent on the effect of the mutation on the SLC4A11 protein, as our work suggests, or the zygosity of the mutation, as suggested by prior studies.

## Supporting information

S1 Table Guide RNA, sequencing and cloning primers used in the generation of a stable *SLC4A11*^-/-^ human corneal endothelial cell (hCEnC) line and site-directed mutagenesis and validation primers used in the generation of stable hCEnC lines expressing mutant *SLC4A11* variant 2 and variant 3. (XLSX)

S1 Fig Immunoblot confirms the absence of SLC4A11 protein expression in CEnC subclones (S11, S17, S21) that underwent *SLC4A11* disruption by CRISPR-Cas9 mediated gene editing. The top row shows Sanger sequencing analysis results by CRISP-ID of each sub-clone and the second row indicates the predicted impact on SLC4A11 protein expression. The third row indicates the actual impact on SLC4A11 protein expression based on the immunoblot of SLC4A11 shown in the fourth row. The fifth row show GAPDH expression as a loading control.
(DOCX)

S2 Fig SLC4A11 protein expression in SLC4A11 V2^WT^, V3^WT^, V2^MU^, V3^MU^ and SLC4A11^-/-^ empty hCEnC lines. SLC4A11 protein expression was detected by capillary immunoblot and results are shown in electropherograms. SLC4A11 (with a calculated molecular weight of ~100 kDa) was detected within broad plateaus that start from ~100kDa to molecular weights above 200kDa. The broad molecular weight range of the detected SLC4A11 protein could potentially be explained by the presence of SLC4A11 glycosylation and/or dimerization. GAPDH expression (blue peaks at the 38–41 kDa range) was also detected for loading controls.
(DOCX)

S3 Fig Fluorescence microscopy images of primary hCEnC. ICC microscopy images showing primary hCEnC fixed with 4% paraformaldehyde and permeabilized with either (A) 0.25% Triton-X or (B) methanol. Immunostaining was performed with an anti-SLC4A11 antibody (Invitrogen) (red, upper-left) and an anti-COX4 antibody (green, upper-right). Lower-left panels show DAPI-stained nuclei (blue) and lower-right panels show merged images. Scale bars: 20 μm.
(DOCX)

S4 Fig Fluorescence microscopy images of primary hCEnC. Immunostaining was performed with custom anti-SLC4A11 antibodies (anti-SLC4A11-M36 antibody in A and B; anti-SLC4A11-V3 antibody in C and D) (red, upper-left panels), an anti-COX4 antibody (green, upper-right panels), and DAPI (blue, lower-left panels). Lower-right panels show merged images. Scale bars: 20 μm.
(DOCX)

S5 Fig Fluorescence microscopy images of SLC4A11 V2^WT^ and V3^WT^ hCEnC. Immunostaining was performed with custom anti-SLC4A11 antibodies (anti-SLC4A11-M36 antibody in A and B; anti-SLC4A11-V3 antibody in C) (red, left panels) and DAPI (blue, middle panels). Right panels show merged images. Scale bars: 20 μm.
(DOCX)

S6 Fig Fluorescence microscopy images of basolateral staining of SLC4A11 in primary hCEnC. Confocal fluorescence microscopy images of primary hCEnC stained with an anti-SLC4A11 antibody (green) and either anti-ZO-1, anti-α-tubulin, anti-COX4, anti-GM130, anti-N-cadherin, or anti-PDI antibodies (red). Apical and basolateral sides of the cell were labeled as empty arrowheads and filled arrowheads on z-axis. Apical and basolateral images are shown in second and third columns to illustrate the basolateral staining patterns of SLC4A11. Scale bars: 5 μm.
(DOCX)
