# Supplementary figures and images for: Investigation of the functional impact of CHED- and FECD4-associated SLC4A11 mutations in human corneal endothelial cells
Source: PLoS One. 2024 Jan 22;19(1):e0296928. doi: 10.1371/journal.pone.0296928 (PMC10802951; doi:10.1371/journal.pone.0296928)

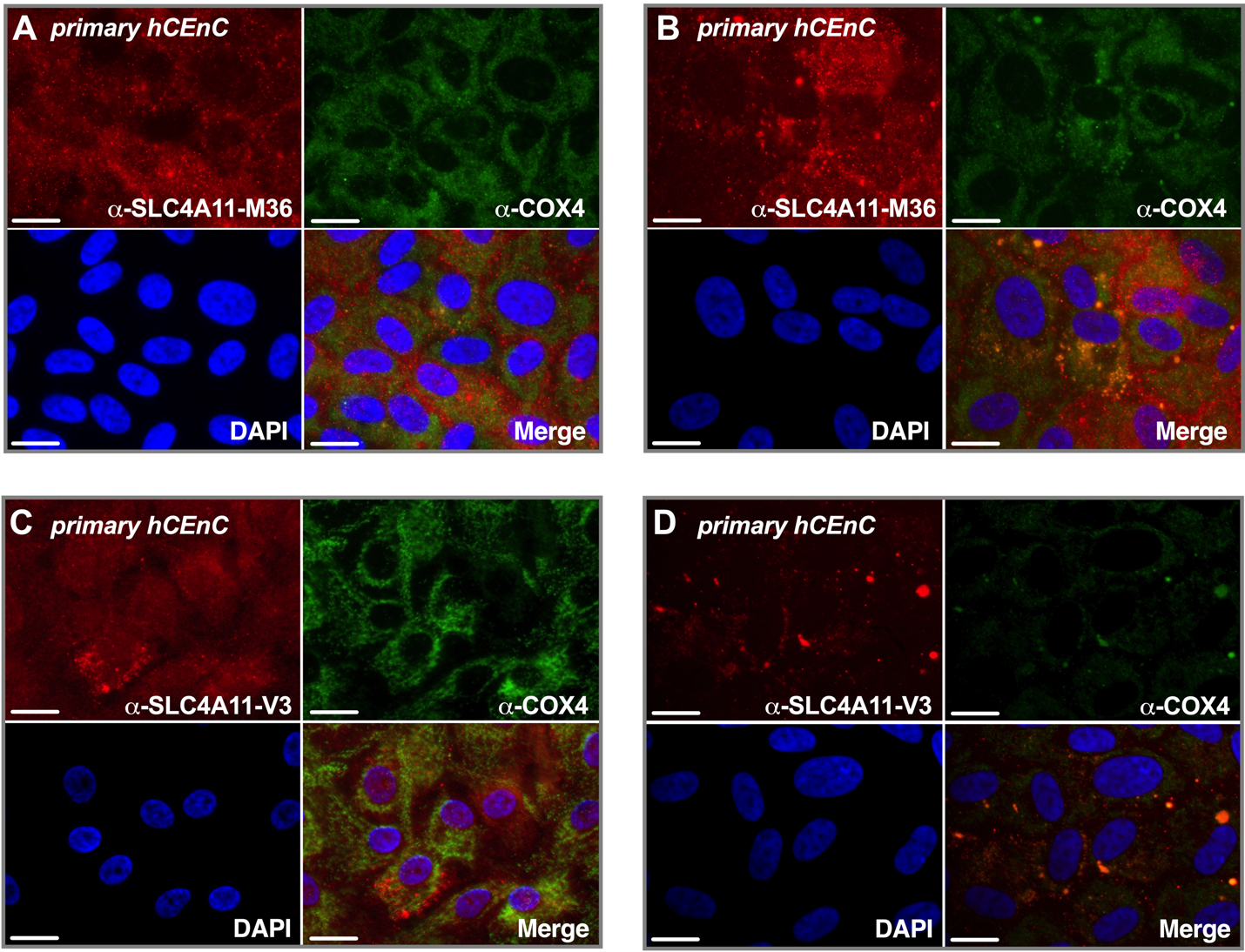

Supplement: S4 Fig — Immunostaining was performed with custom anti-SLC4A11 antibodies (anti-SLC4A11-M36 antibody in A and B; anti-SLC4A11-V3 antibody in C and D) (red, upper-left panels), an anti-COX4 antibody (green, upper-right panels), and DAPI (blue, lower-left panels). Lower-right panels show merged images. Scale bars: 20 μm. (DOCX) [file pone.0296928.s005.docx]

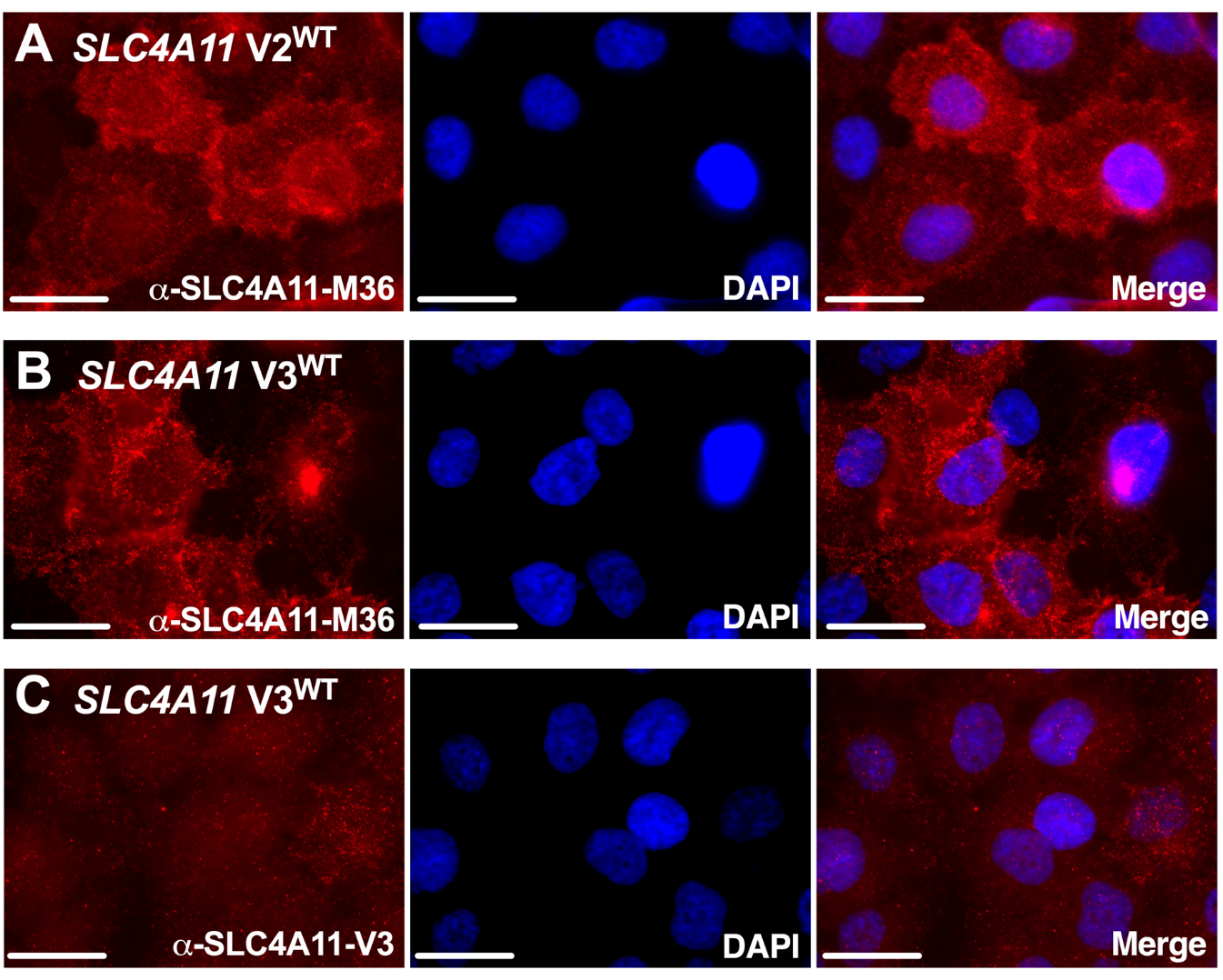

Supplement: S5 Fig — Immunostaining was performed with custom anti-SLC4A11 antibodies (anti-SLC4A11-M36 antibody in A and B; anti-SLC4A11-V3 antibody in C) (red, left panels) and DAPI (blue, middle panels). Right panels show merged images. Scale bars: 20 μm. (DOCX) [file pone.0296928.s006.docx]

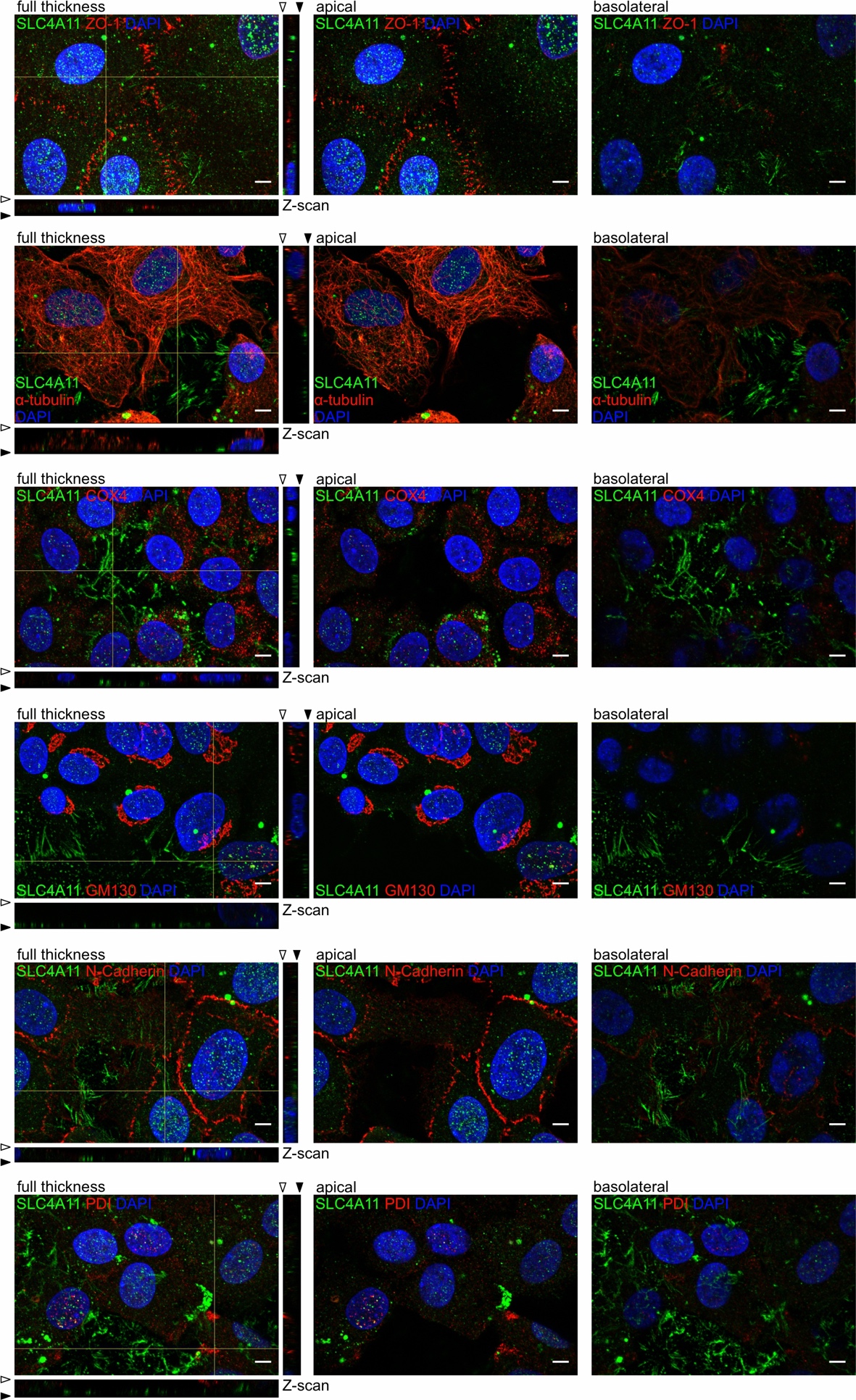

Supplement: S6 Fig — Confocal fluorescence microscopy images of primary hCEnC stained with an anti-SLC4A11 antibody (green) and either anti-ZO-1, anti-α-tubulin, anti-COX4, anti-GM130, anti-N-cadherin, or anti-PDI antibodies (red). Apical and basolateral sides of the cell were labeled as empty arrowheads and filled arrowheads on z-axis. Apical and basolateral images are shown in second and third columns to illustrate the basolateral staining patterns of SLC4A11. Scale bars: 5 μm. (DOCX) [file pone.0296928.s007.docx]
