# Supplementary material for: Investigation of the functional impact of CHED- and FECD4-associated SLC4A11 mutations in human corneal endothelial cells
Source: PLoS One. 2024 Jan 22;19(1):e0296928. doi: 10.1371/journal.pone.0296928 (PMC10802951; doi:10.1371/journal.pone.0296928)
Supplement: Supplementary file 3 [file pone.0296928.s002.docx]

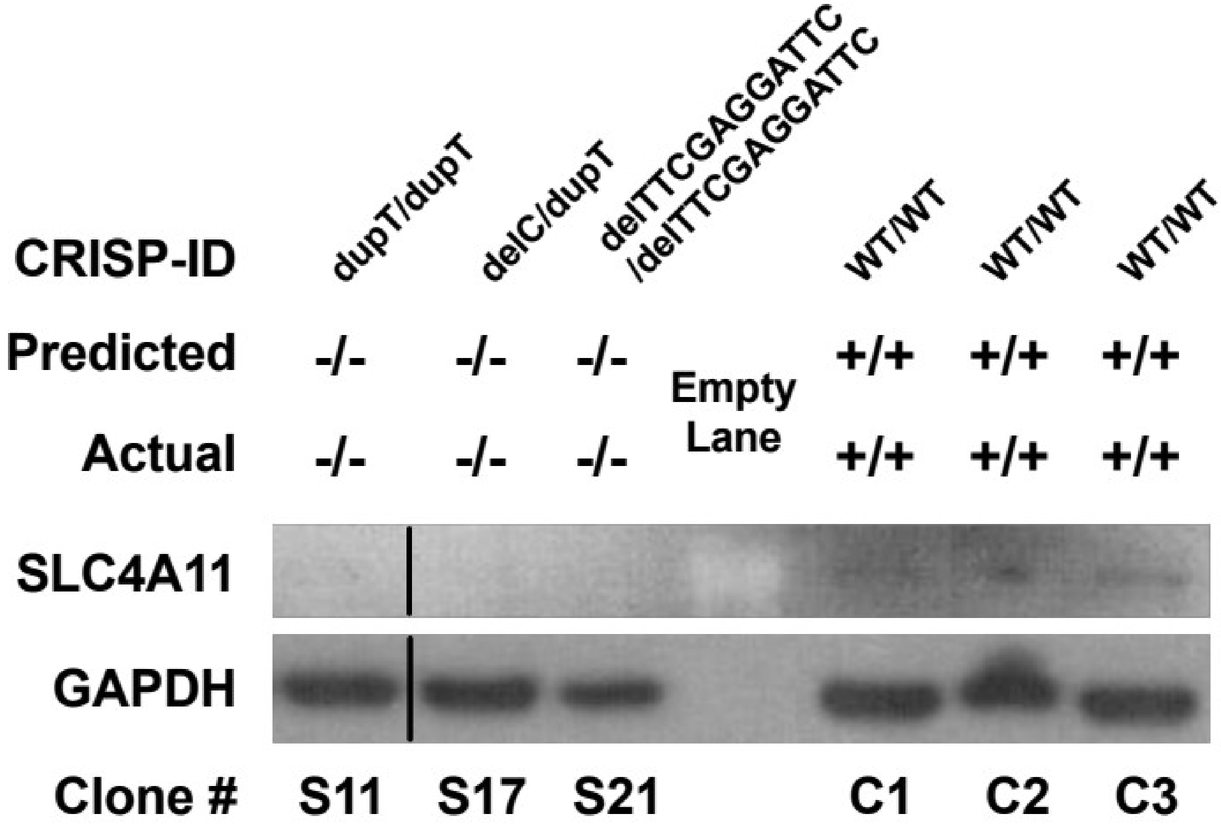

CRISP-ID
dupT/dupT
delC/dupT
delTTCGAGGATTC
/delTTCGAGGATTC
WT/WT
WT/WT
WT/WT
Predicted
-/-
-/-
-/-
Empty
Lane
+/+
+/+
+/+
Actual
-/-
-/-
-/-
+/+
+/+
+/+
SLC4A11
GAPDH
Clone #
S11
S17
S21
C1
C2
C3
