# Supplementary material for: Investigation of the functional impact of CHED- and FECD4-associated SLC4A11 mutations in human corneal endothelial cells
Source: PLoS One. 2024 Jan 22;19(1):e0296928. doi: 10.1371/journal.pone.0296928 (PMC10802951; doi:10.1371/journal.pone.0296928)
Supplement: Supplementary file 4 [file pone.0296928.s003.docx]

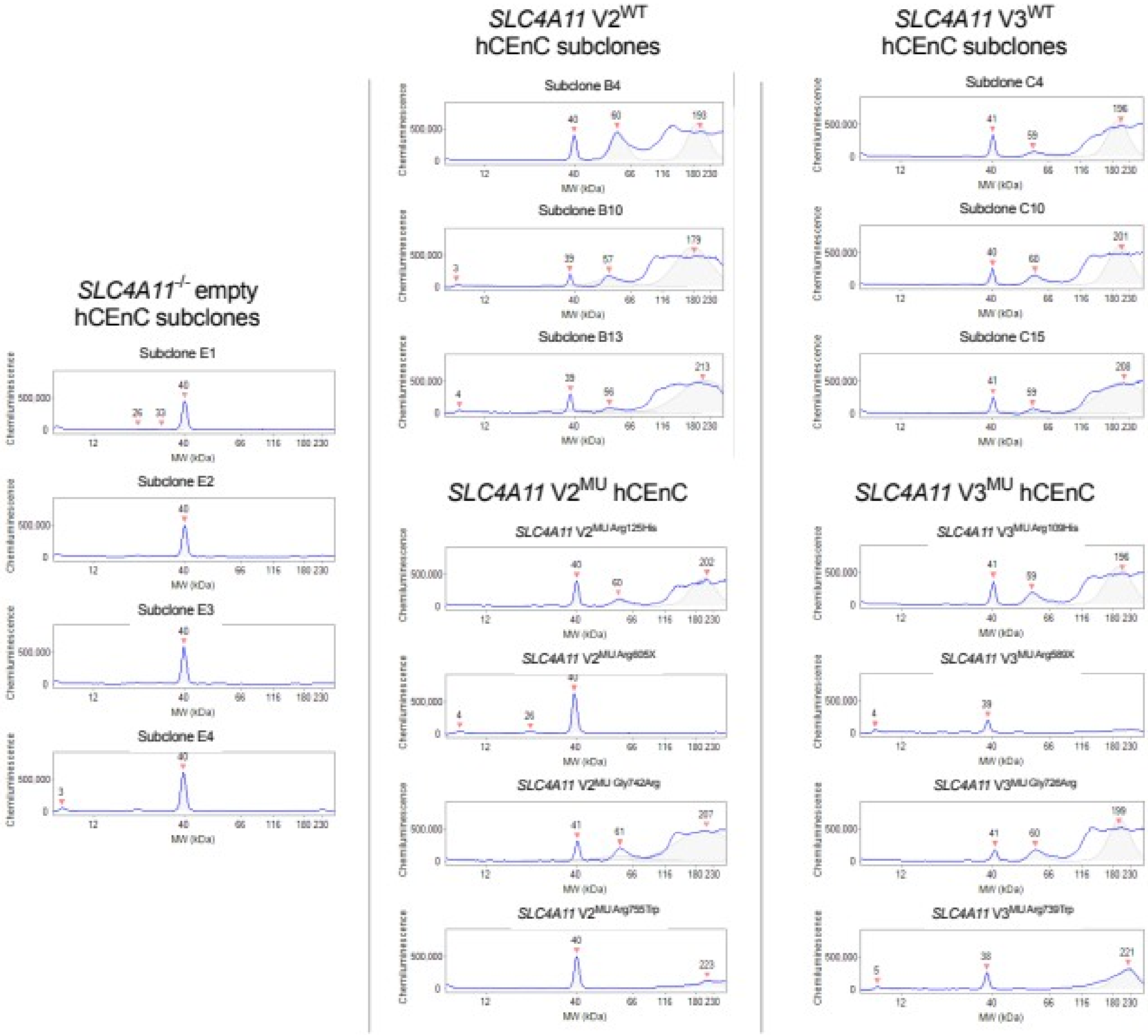

SLC4A11 V2WT hCEnC subclones
SLC4A11 V3WT hCEnC subclones
SLC4A11-/- empty hCEnC subclones
Subclone E1
Subclone E2
Subclone E3
Subclone E4
Subclone B4
Subclone B10
Subclone B13
Subclone C4
Subclone C10
Subclone C15
SLC4A11 V2MU hCEnC
SLC4A11 V3MU hCEnC
SLC4A11 V2MU Arg125His
SLC4A11 V2MU Arg605X
SLC4A11 V2MU Gly742Arg
SLC4A11 V2MU Arg755Trp
SLC4A11 V3MU Arg109His
SLC4A11 V3MU Gly726Arg
SLC4A11 V3MU Arg739Trp
Chemiluminescence
MW (kDa)
