# Supplementary material for: Investigation of the functional impact of CHED- and FECD4-associated SLC4A11 mutations in human corneal endothelial cells
Source: PLoS One. 2024 Jan 22;19(1):e0296928. doi: 10.1371/journal.pone.0296928 (PMC10802951; doi:10.1371/journal.pone.0296928)
Supplement: Supplementary file 5 [file pone.0296928.s004.docx]

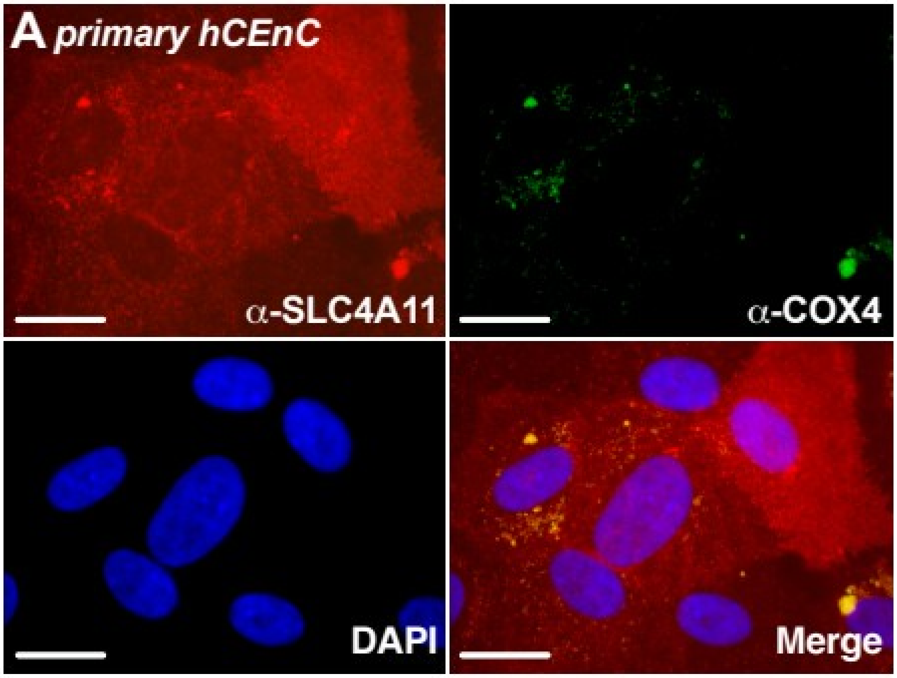
A primary hCEnC
α-SLC4A11
α-COX4
DAPI
Merge

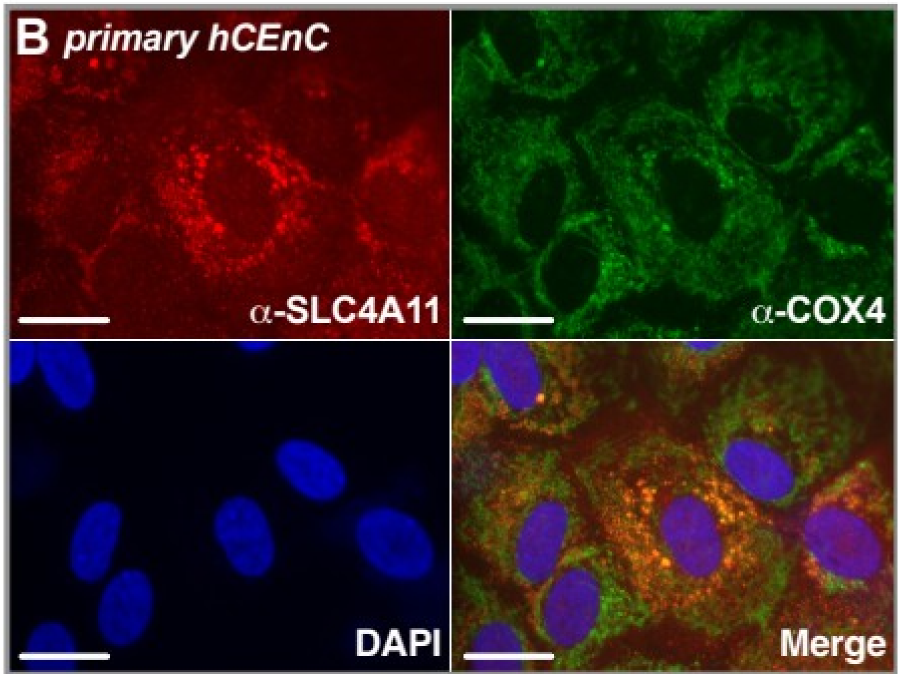
B primary hCEnC
α-SLC4A11
α-COX4
DAPI
Merge
